# SARS-CoV-2 NSP5 antagonizes MHC II expression by subverting histone deacetylase 2

**DOI:** 10.1242/jcs.262172

**Published:** 2024-05-22

**Authors:** Nima Taefehshokr, Alex Lac, Angela M. Vrieze, Brandon H. Dickson, Peter N. Guo, Catherine Jung, Eoin N. Blythe, Corby Fink, Amena Aktar, Jimmy D. Dikeakos, Gregory A. Dekaban, Bryan Heit

**Affiliations:** ^1^ Department of Microbiology and Immunology, and the Western Infection, Immunity and Inflammation Centre, The University of Western Ontario, London, Ontario, Canada N6A 5C1; ^2^ Robarts Research Institute, London, Ontario, Canada N6A 3K7

**Keywords:** SARS-CoV-2, NSP5, MHC II, CIITA, Antigen presentation, Dendritic cells, Macrophages, IRF3

## Abstract

SARS-CoV-2 interferes with antigen presentation by downregulating major histocompatibility complex (MHC) II on antigen-presenting cells, but the mechanism mediating this process is unelucidated. Herein, analysis of protein and gene expression in human antigen-presenting cells reveals that MHC II is downregulated by the SARS-CoV-2 main protease, NSP5. This suppression of MHC II expression occurs via decreased expression of the MHC II regulatory protein CIITA. CIITA downregulation is independent of the proteolytic activity of NSP5, and rather, NSP5 delivers HDAC2 to the transcription factor IRF3 at an IRF-binding site within the CIITA promoter. Here, HDAC2 deacetylates and inactivates the CIITA promoter. This loss of CIITA expression prevents further expression of MHC II, with this suppression alleviated by ectopic expression of CIITA or knockdown of HDAC2. These results identify a mechanism by which SARS-CoV-2 limits MHC II expression, thereby delaying or weakening the subsequent adaptive immune response.

## Teaser

**Summary:** SARS-CoV-2 limits T cells activation by reducing MHC II expression on antigen-presenting cells. This suppression of MHC II occurs via inactivation of the CIITA promoter by a complex of viral NSP5 and host IRF3 and HDAC2.

## INTRODUCTION

Severe acute respiratory syndrome coronavirus 2 (SARS-CoV-2) rapidly became a leading global cause of morbidity and mortality, with current vaccination and antiviral strategies greatly reducing mortality (Canevari et al., 2024; Català et al., 2024; Tan et al., 2020; Zhu et al., 2020a). This high virulence is due, in part, to multiple mechanisms enabling SARS-CoV-2 to evade and alter host immune responses, thereby delaying viral clearance and prolonging the infection period (Taefehshokr et al., 2020; Zhu et al., 2020a). Humoral immunity against SARS-CoV-2 wanes quickly, allowing for repeat infections, an issue further compounded by the emergence of new SARS-CoV-2 variants (Bergwerk et al., 2021; Kuhlmann et al., 2022; Townsend et al., 2021). Therefore, understanding the immunoevasion mechanisms of SARS-CoV-2 is important to better understand this disease and to develop better-targeted treatments and vaccines.

Although some subsets of professional antigen-presenting cells (pAPCs), including macrophages and dendritic cells (DCs) do not express ACE2 – the canonical receptor for SARS-CoV-2 viral entry – these cells can become infected via Fc receptor-dependent phagocytosis of antibody-opsonized virions and potentially through the efferocytosis of SARS-CoV-2 infected apoptotic cells (Grant et al., 2021; Junqueira et al., 2022; Pontelli et al., 2022; Salina et al., 2022; Sefik et al., 2022; Wendisch et al., 2021; Zhou et al., 2020). Although SARS-CoV-2 is unable to establish a productive infection in macrophages or DCs, viral early genes are expressed in these cells and drive a multi-pronged immunoevasion response. Firstly, the expression of pro-inflammatory cytokines is induced and contributes to the cytokine storm (Sefik et al., 2022). This cytokine response is typified by high circulating levels of IL-2, IL-6, IL-7, IL-8, IL-10, IP-10 (also known as CXCL10), G-CSF (CSF3R), MCP-1 (CCL2), MIP1-α (CCL3) and TNF-α (Del Valle et al., 2020; Huang et al., 2020; Song et al., 2020). Although this drives a potent inflammatory response, the cytokine profile is more typical of bacterial infections and promotes both natural killer (NK) cell exhaustion and reduced NK cell cytotoxicity, thereby producing a non-productive innate immune response that can exacerbate tissue damage (Lassen et al., 2010; Mazzoni et al., 2020). Secondly, SARS-CoV-2 suppresses the antiviral interferon (IFN) pathway, reducing the production of type I and type II IFNs. This suppression is driven by ORF6, which sequesters inactive signal transducer and activator of transcription 1 (STAT1) and STAT2 in the cytosol, thereby preventing their nuclear translocation and blocking the primary signaling pathway that initiates antiviral IFN responses (Miorin et al., 2020). Moreover, membrane protein and non-structural protein 13 further inhibit IFN-I production by degrading TANK-binding kinase 1 (Sui et al., 2021, 2022). Thirdly, infected macrophages and DCs often die, resulting in long-term depletion of some subsets of immune cells (Chang et al., 2022). Fourthly, SARS-CoV-2 directly suppresses antigen presentation by major histocompatibility complex (MHC) class I (MHC I). This suppression occurs through ORF8-mediated redirection of MHC I trafficking to lysosomes where it is degraded (Zhang et al., 2021) and by ORF6-mediated inactivation of MHC I transcription. This suppression of MHC I expression limits the killing of SARS-CoV-2 infected cells by CD8^+^ T cells (Yoo et al., 2021). Finally, infection of alveolar DCs reduces their ability to migrate to draining lymph nodes and suppresses expression of MHC II and the class II transcriptional activator (CIITA) required for MHC II expression (Saichi et al., 2021). This suppression of MHC II occurs across a range of pAPCs in SARS-CoV-2-infected individuals and in cells infected *in vitro* (Giamarellos-Bourboulis et al., 2020; Munnur et al., 2021; Wilk et al., 2020). Moreover, some non-professional APCs express MHC II in response to infection – including the type II alveolar epithelial cells that are a primary target of SARS-CoV-2 infection (Munster et al., 2020; Ziegler et al., 2020). However, the mechanism through which SARS-CoV-2 downregulates MHC II has remained elusive.

MHC II expression is driven by type II interferon signaling (Steimle et al., 1994). Activation of the IFN-γ receptor complex leads to the structural rearrangements in the receptor complex and activation of Janus kinase 1 and 2 (JAK1 and JAK2, collectively JAK1/2). JAK1/2 in turn phosphorylates STAT1, which then dimerizes and translocates to the nucleus where it induces transcription of IFN-γ-inducible genes (Hu and Ivashkiv, 2009). In parallel, Toll-like receptors and viral sensors such as cGas–STING activate interferon regulatory factors -1 and -3 (IRF1/3), which induce the expression of CIITA in cooperation with STAT1 (Muhlethaler-Mottet et al., 1998; Zhang et al., 2013). CIITA, via its intrinsic acetyltransferase activity, then acetylates histones at the MHC II promoter, decondensing the chromatin (Beresford and Boss, 2001). Once the chromatin is opened, CIITA and regulatory factor X5 (RFX5) form an enhanceosome complex on the MHC II promoter which recruits and activates additional transcription factors that induce transcription of MHC II (Masternak et al., 2000). Although inhibition of STAT1 nuclear import by SARS-CoV-2 ORF6 might account for some inhibition of MHC II expression (Miorin et al., 2020), tissue-resident DCs constitutively express significant amounts of CIITA (Landmann et al., 2001), whereas ORF6 expression is limited until 12–16 h post infection (Kim et al., 2021); this is more than sufficient time to induce presentation of SARS-CoV-2 antigens following infection. Moreover, IFN-independent mechanisms can drive MHC II expression in macrophages and DCs (Lee et al., 2006; Zhou et al., 1997). Therefore, a more direct form of MHC II suppression is likely invoked by SARS-CoV-2.

A crucial regulator of MHC II expression is histone deacetylase 2 (HDAC2), which suppresses the expression of CIITA and MHC II through histone deacetylation within their promoters (Gialitakis et al., 2006). Gordon et al. mapped the SARS-CoV-2 protein interactome, identifying non-structural protein 5 (NSP5) as an HDAC2 interactor, and identified a putative NSP5 cleavage site near the nuclear localization signal (NLS) of HDAC2 (Gordon et al., 2020). NSP5 – also known as main protease and 3-chymotrypsin-like protease – is translated as part of the polyprotein expressed early after viral entry. Once this polyprotein is translated, NSP5 cleaves it into 11 individual proteins to form a complex that translates the full viral RNA, thereby reproducing the viral genome (Jin et al., 2020). Through interactions with HDAC2, NSP5 mediates the epigenetic reprogramming of infected cells, which in pAPCs might include suppression of MHC II expression. Indeed, epigenetic changes are required for SARS-CoV-2 reproduction (Chlamydas et al., 2021; Salgado-Albarrán et al., 2021), and similar epigenetic reprogramming is known to suppress MHC II expression in Middle East respiratory syndrome coronavirus (MERS-CoV) (Josset et al., 2013). Therefore, we tested the hypothesis that SARS-CoV-2 NSP5 inhibits MHC II expression through HDAC2.

## RESULTS

### SARS-CoV-2 NSP5 downregulates MHC II in pAPCs

**Fig. 1.**
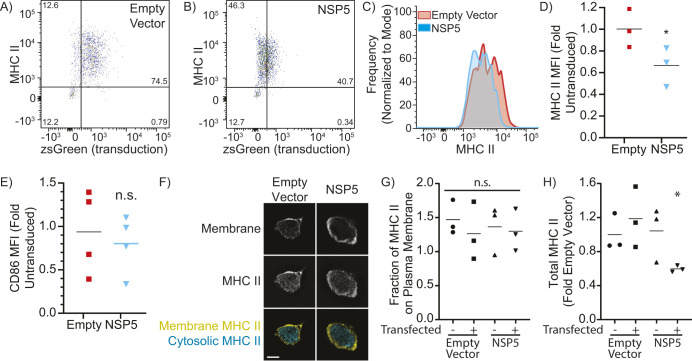
**NSP5 suppresses MHC II in primary human monocyte-derived DCs.** moDCs were transduced with lentiviral vectors lacking a transgene (empty vector) or bearing NSP5, with both vectors containing an IRES-zsGreen marker. (A,B) Representative flow cytometry plots showing cell surface MHC II expression and transduction (zsGreen^+^) of moDCs transduced with an empty vector control (A) or NSP5-expressing lentiviral vector (B). (C) Histogram of cell surface MHC II expression levels on moDCs transduced with an empty vector (red) or NSP5-expressing vector (cyan). (D,E) Quantification of cell surface MHC II (D) and CD86 (E) in moDCs transduced with either an empty (Empty) or NSP5-expressing (NSP5) lentiviral vector. The mean fluorescence intensity (MFI) is normalized to the MFI of the non-transduced cells in the empty vector condition. (F) Z-slice through a macrophage stained for MHC II and the plasma membrane, showing the segmentation of vesicular and cytosolic versus surface (membrane) MHC II. Scale bar: 10 µm. (G,H) Quantification of the fraction of MHC II on the plasma membrane (G) and total cellular MHC II (H) in macrophages that have been transfected either with an empty or NSP5-expressing vector, comparing non-transfected (zsGreen-negative) to transfected (zsGreen-positive) cells in both conditions. Data is representative of, or quantifies, a minimum of three independent experiments. Line in D, E, G and H shows the mean. *P<0.05; n.s., not significant (*P*>0.05) (Kruskal–Wallace test with Dunn correction compared to non-transfected empty vector).

As deletion or inactivation of NSP5 abrogates productive infection by SARS-CoV-2 (Hegyi and Ziebuhr, 2002; Jin et al., 2020; Lee et al., 2022), ectopic expression of NSP5 was used to determine NSP5's impact on MHC II expression in primary human monocyte-derived DCs (moDCs). Flow cytometry was used to quantify the total surface expression of MHC II on transduced (zsGreen^+^) moDCs (Fig. 1A,B; Fig. S1A–C), with NSP5 expression reducing the cell surface expression of MHC II to an extent similar to that observed in individuals with SARS-CoV-2 (30–50% reduction, Fig. 1C,D; Giamarellos-Bourboulis et al., 2020; Munnur et al., 2021; Wilk et al., 2020). This downregulation was not a general suppression of the MHC II presentation system, as expression of the co-stimulatory molecule CD86 was not affected by NSP5 (Fig. 1E). Some pathogens such as human Cytomegalovirus reduce their immunogenicity by diverting the intracellular trafficking of MHC II away from the cell surface (Stumptner-Cuvelette et al., 2001). To test this possibility, J774.2 macrophages were transduced with NSP5-expressing or empty vectors, and the plasma membrane was labeled with wheat-germ agglutinin (WGA), followed by labeling for total cellular MHC II. Three-dimensional reconstructions of these cells were used to differentiate between cytosolic and vesicular MHC II and cell-surface MHC II (Fig. 1F). Quantification of these micrographs revealed no changes in the proportion of MHC II localized to the cell surface versus intracellular vacuoles in NSP5-expressing cells (Fig. 1G) but did identify the same decrease in MHC II expression that was observed with flow cytometry (Fig. 1D,H). This indicates that NSP5 does not affect the trafficking of MHC II to the cell surface, but rather decreases its overall expression. Moreover, this effect was only observed in NSP5-expressing cells, but not in neighboring non-transduced cells, indicating that this effect is cell intrinsic and not due to NSP5-induced changes in the expression of cytokines or other secreted factors.

### NSP5 suppresses CIITA and MHC II transcription

**Fig. 2.**
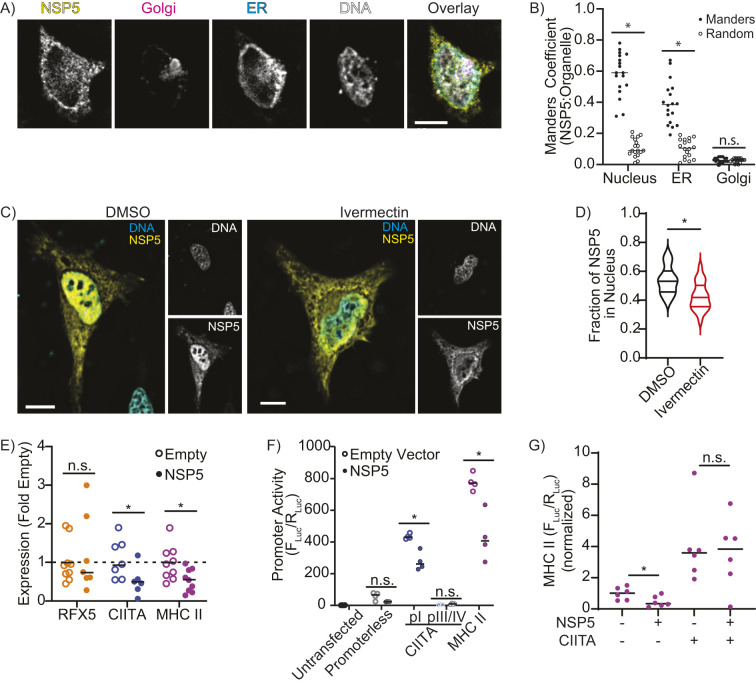
**NSP5 suppresses CIITA and MHC II expression.** (A,B) Fluorescence *Z*-projection (A) and Manders’ colocalization analysis (B) quantifying the proportion of NSP5 colocalized with the nucleus for HeLa cells co-transfected with NSP5–FLAG (yellow), TGN46–mCherry (Golgi, magenta), KDEL–eGFP (ER, cyan) and stained with Hoechst (DNA, gray). Manders colocalization analysis compares the fraction of NSP5 colocalized with the nucleus, ER and Golgi (Manders) to the Manders’ ratio from the same image when the NSP5 image was randomized (Random). (C,D) Fluorescent *z*-projections (C) and quantification (D, violin plot highlighting median and quartiles) of the fraction of NSP5 in the nucleus of vehicle-treated (DMSO) versus ivermectin-treated HeLa cells expressing NSP5–FLAG (yellow) and stained for DNA with Hoechst (cyan). (E) RT-qPCR quantification of RFX5, CIITA and MHC II mRNA levels in moDCs transduced with empty or NSP5-expressing lentiviral vectors. (F) Quantification of the promoter activity of the CIITA pI, CIITA pIII/IV, and MHC II promoters using a dual-luciferase assay in RAW 264.7 macrophages co-transfected with empty or NSP5-expressing lentiviral vectors. (G) Quantification of MHC II promoter activity in RAW 264.7 macrophages co-transfected with CIITA and NSP5. (−) indicates the sample was transfected with empty vector rather than CIITA or NSP5. *F*_luc_/*R*_luc_ was normalized to the value of cells transfected with an empty vector. Images are representative of a minimum of 30 cells captured across three independent experiments. *n*=minimum of 3. Line in B and E–G shows the mean. *P<0.05; n.s., not significant (*P*>0.05) [paired two-tailed *t*-test (B) or Mann–Whitney test (D–G) compared to Random (B), DMSO (D), or Empty Vector (E–G)]. Scale bars: 10 µm.

Next, the subcellular localization of NSP5 was determined to identify potential mechanisms accounting for the downregulation of MHC II. Quantitative microscopy of HeLa cells expressing NSP5–FLAG, the endoplasmic reticulum (ER) marker KDEL–GFP, the Golgi marker GalT–mCherry and with the nuclei stained with Hoechst 33258 determined that approximately half of the cellular NSP5 was localized to the nucleus, with the remainder associated with the ER (Fig. 2A,B). The nuclear localization of NSP5 was further confirmed by pharmacologically blocking importin-mediated nuclear transport with ivermectin (Fig. 2C,D), but the impact of blocking nuclear import on NSP5 activity could not be determined as blocking nuclear import also blocked CIITA expression (Fig. S1D), likely as CIITA function requires the nuclear import of STAT1 (Cressman et al., 1999; Krämer et al., 2009; Piskurich et al., 1998). Although localization to the ER is consistent with the known role of NSP5 in forming the viral replication complex (Santerre et al., 2021), the role of nuclear NSP5 remains unclear. Unexpectedly, bioinformatic analysis of NSP5 failed to identify either a classical or bipartite NLS or a nuclear export signal. This suggests that NSP5 might be carried into the nucleus through interactions with other cellular proteins, similar to what occurs for the hepatitis delta antigen (Xia et al., 1992).

The nuclear localization of NSP5 suggests that downregulation of MHC II occurs via a transcriptional mechanism. To test this hypothesis, real-time quantitative RT-PCR (RT-qPCR) was used to quantify the mRNA levels of MHC II, and of RFX5 and CIITA – two transcription factors that act as master regulators of MHC II expression. Interestingly, although RFX5 expression was unchanged, NSP5 significantly downregulated the expression of CIITA and MHC II relative to the levels normally found in resting moDCs (Fig. 2E). In humans, CIITA is transcribed from three separate promoters: pI, which drives expression in myeloid cells; pIII, which drives expression in lymphoid cells; and pIV, which drives IFN-γ-induced expression in non-immune cells, such as epithelia (Morris et al., 2002; Zinzow-Kramer et al., 2012). Dual-luciferase reporters of the MHC II promoter, the CIITA pI promoter and the region containing the CIITA pIII and pIV promoters (hereafter CIITA pIII/IV) were used to quantify the impact of NSP5 expression on the activity of these promoters in macrophages (Fig. S1E). NSP5 expression strongly suppressed both the MHC II promoter and the CIITA pI promoter, whereas the CIITA pIII/IV promoters were minimally active, producing insufficient signal to observe any effect of NSP5 (Fig. 2F). MHC II transcription is dependent on CIITA, therefore this suppression of MHC II expression might be due to NSP5-mediated suppression of CIITA expression, or might be a product of NSP5 suppression of both the CIITA and MHC II promoters. To differentiate between these possibilities, MHC II promoter activity was quantified in cells ectopically expressing CIITA and NSP5 (Fig. 2G). CIITA expression greatly increased MHC II promoter activity, with co-expression of NSP5 not affecting MHC II promoter activity in the presence of ectopically expressed CIITA. Importantly, SARS-CoV-2 infection of A549 cells produced similar levels of NSP5 expression, as does our lentiviral transduction model (Fig. S1F). Although viral infections normally induce MHC II and CIITA expression in A549 cells (Guo et al., 2015), neither SARS-CoV-2 infection nor transduction with an empty lentiviral vector, induced expression of either gene (Fig. S1G). These data indicate that NSP5 likely functions by suppressing the transcription of CIITA, with the resulting absence of CIITA then limiting MHC II expression.

### NSP5-mediated suppression of MHC II expression is dependent on HDAC2

**Fig. 3.**
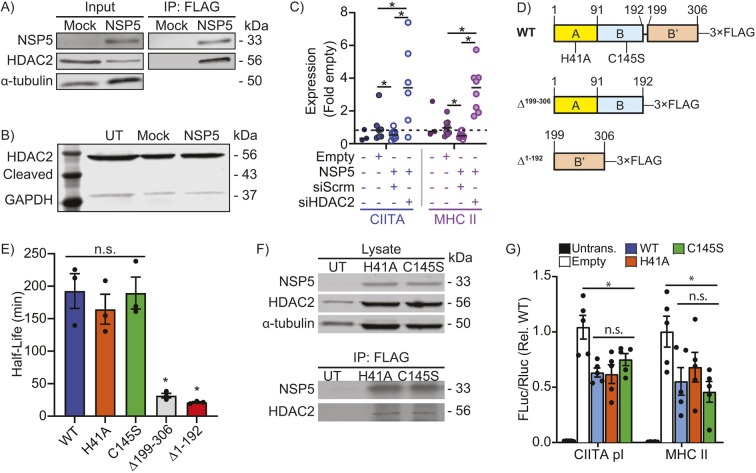
**NSP5 requires HDAC2 to suppress MHC II expression.** (A) Co-immunoprecipitation (IP) of HDAC2 with NSP5–FLAG from primary human moDCs transduced with empty (Mock) or NSP5–FLAG-expressing lentiviral vectors. Input, 5%. (B) Absence of detectable cleavage of endogenous HDAC2 in cells ectopically expressing NSP5. Cells are either untreated (UT), transfected with an empty vector (Mock) or transfected with NSP5, and the blots stained for endogenous HDAC2 and GAPDH. The expected size of NSP5-cleaved HDAC2 is 43.7 kDa. (C) RT-qPCR quantification of the impact of scrambled (siScrm) or HDAC2-targeting (siHDAC2) siRNA on CIITA and MHC II mRNA levels in primary human moDCs transduced with empty or NSP5-expressing lentiviral vectors. (D) Protease-inactivating (H41A and C145S) point-mutants, and deletion of the catalytic (A/B, Δ1–192) and ligand-stabilizing (B′, Δ199–306) domains were generated to assay the roles of these sites in NSP5 activity. WT, wild type. (E) Half-life of NSP5 and the H41A, C145S, Δ1–192 and Δ199–306 mutants, as quantified by NSP5 densitometry in cycloheximide-treated cells. (F) Immunoprecipitation of HDAC2 with NSP5^H41A^ and NSP5^C145S^ mutants. (G) Dual-luciferase assay quantification of CIITA pI and MHC II promoter activity in RAW264.7 macrophages that were either untransfected (Untrans.) or co-transfected with the CIITA or MHC II luciferase constructs plus either the empty vector (Empty), or with one of wild-type NSP5 (WT), NSP5^H41A^ or NSP5^C145S^ vectors. Data is normalized to Empty. For all panels, *n*=3–5. Results in E,G are mean±s.e.m. *P<0.05; n.s., not significant (*P*>0.05) [Kruskal–Wallis test with Dunn correction].

MHC II expression is dependent on CIITA, with MHC II expression beginning with CIITA binding to distal enhancers located 5′ to the MHC II promoter (Krawczyk et al., 2004). This is followed by CIITA-mediated acetylation of histones and transcription factors within the core MHC II promoter, forming an enhanceosome complex that initiates MHC II transcription (Raval et al., 2001; Soe et al., 2013). CIITA expression is induced by IFN-γ through the transcription factors STAT1 and IRF1, and by Toll-like receptor (TLR) and IL-1 family cytokines via the transcription factor IRF3 (Mantegazza et al., 2012, 2014; Muhlethaler-Mottet et al., 1998), and is negatively regulated by HDAC2-mediated promoter deacetylation (Kong et al., 2009). Crucially, SARS-CoV-2 NSP5 has been shown to interact with HDAC2, suggesting that NSP5 might use HDAC2 to silence the CIITA promoter (Gordon et al., 2020). Consistent with this model, endogenous HDAC2 co-immunoprecipitated with FLAG-tagged NSP5 in an anti-FLAG immunoprecipitation (Fig. 3A). This ectopically expressed NSP5 was confirmed to be proteolytically active using both a FRET reporter and immunoblotting (Fig. S2). Given this proteolytic activity and prior observations that some HDACs undergo proteolysis-mediated activation (Paroni et al., 2004), HDAC2 immunoblotting was used to detect NSP5-mediated HDAC2 cleavage. This failed to detect the 43 kDa cleavage fragment that would result from NSP5 proteolysis at the predicted cleavage site in HDAC2 (Fig. 3B). To confirm this observation, and to test the possibility that HDAC2 might be cleaved at a site too near to the N- or C-terminus to be detected by immunoblotting, HDAC2 cleavage was quantified using a FRET-based HDAC2 intramolecular cleavage probe (Fig. S3A–C). No change in FRET efficiency was observed between empty-vector- and NSP5-transfected cells, confirming that HDAC2 is not cleaved by NSP5. Knockdown of HDAC2 (Fig. S3D) had a profound restorative effect on CIITA and MHC II mRNA levels, with HDAC2 knockdown not only reversing – but increasing above baseline – expression of both genes (Fig. 3C). NSP5 consists of a proteolytic domain formed by the interface of the globular A and B domains, which positions two key catalytic residues (H41 and C145) in a binding cleft formed between the two domains. The C-terminal B′ chain folds over this cleft, coordinating the substrate and three water molecules within the active site (Lee et al., 2020). The catalytic site of NSP5 was inactivated by generating NSP5^H41A^ and NSP5^C145S^ point mutants, and deletions of the proteolytic (NSP5^Δ1-192^) and B′ domains (NSP5^Δ199-306^) were generated (Fig. 3D). Unfortunately, the deletion mutants were unstable and could not be expressed at levels amenable to further experimentation (Fig. 3E; Fig. S3E,F). As expected, both point mutants lost their proteolytic activity (Fig. S2D,E), but did not lose their binding capacity for HDAC2 (Fig. 3F) or their ability to suppress CIITA and MHC II promoter activity (Fig. 3G).

### NSP5 induces deacetylation of the CIITA and MHC II promoters

**Fig. 4.**
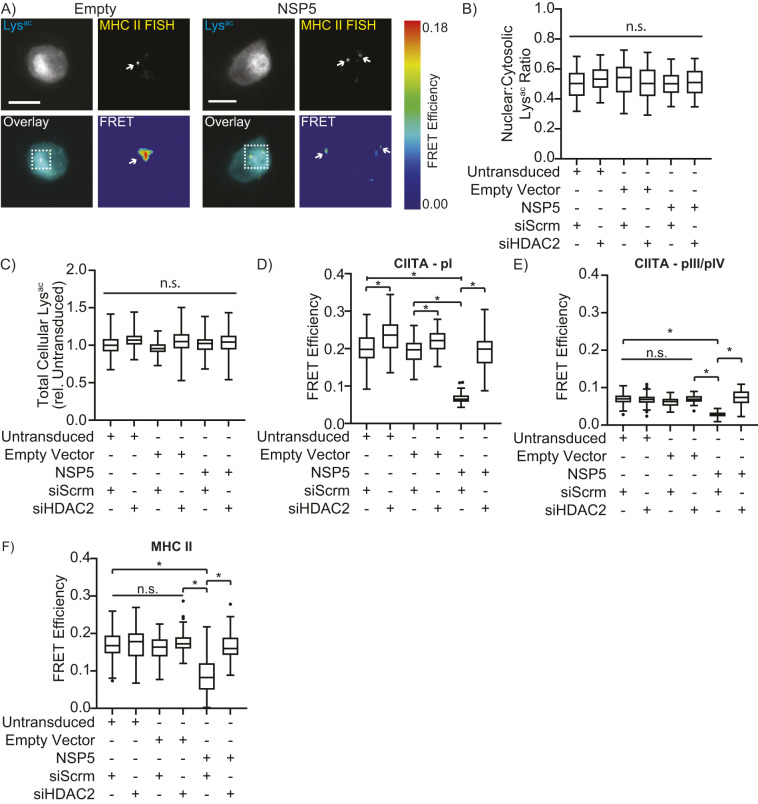
**NSP5 modulates acetylation of the CIITA and MHC II promoters in human macrophages.** (A) Representative micrograph of primary human macrophages transduced with empty or NSP5-expressing lentiviral vectors. Cells were stained for acetyl-lysine (Lys^ac^, cyan) and with ATTO647N-labeled MHC II FISH probes (yellow), with the FRET signal between the Lys^ac^ and FISH probes within the boxed area shown in the FRET panel. Arrows highlight individual FISH probes (top) and the cosponsoring FRET signal (bottom). Scale bars: 10 µm. (B,C) Quantification of the nuclear:cytosolic acetyl-lysine (Lys^ac^) distribution (B) and total cellular acetyl-lysine content (C) in primary human macrophages transduced with empty or NSP5-expressing lentiviral vectors and treated with scrambled (siScrm) or HDAC2-targeting (siHDAC2) siRNAs. ‘Untransduced’ quantifies macrophages that did not take up the empty or NSP5-expressing lentiviral vector. (D–F) Quantification of the acetylation of the CIITA pI (D), CIITA pIII/IV (E) and MHC II (F) promoters, as quantified by the FRET efficiency between Cy3-labeled anti-acetyl-lysine staining and ATTO647N-stained FISH probes in the same cells used for quantification of B and C. Data are representative of (A) or quantify (B–F) three independent experiments, and are plotted as interquartile range with median (box and line) with whiskers representing the 5–95th percentiles. *P<0.05; n.s., not significant (Kruskal–Wallis test with Dunn correction). Horizontal bars over columns indicate the statistical significance for all groups beneath; brackets indicate the statistical significance between the groups below the bracket arms.

As the suppression of MHC II required both NSP5 and HDAC2, NSP5 likely modulates histone acetylation at the CIITA or MHC II promoters. Conventional ChIP was not possible due to the limited (30–50%) efficiency of transduction. Instead, fluorescence *in situ* hybridization-fluorescence resonance energy transfer (FISH-FRET) microscopy was used to compare acetylation of the MHC II and CIITA promoters by detecting Cy3-labeled acetyl-lysine residues that were within 10 Å of ATTO647-N-labeled FISH probes specific to the MHC II promoter, to the pI promoter of CIITA or to CIITA pIII/IV (Fig. S1E). Before imaging, cells were transduced with empty or NSP5-expressing vectors and treated with either scrambled or HDAC2-targeting siRNAs. In all cells, acetylated-lysine staining was concentrated in the nucleus, with weaker staining in the cytosol, and with one or two FISH probes in-focus within each nucleus (Fig. 4A). Neither NSP5 expression nor HDAC2 depletion altered the quantity or distribution of cellular acetyl-lysine staining (Fig. 4B,C), indicating that neither NSP5 expression nor HDAC2 knockdown globally affected lysine acetylation. Both non-transduced cells and cells transduced with an empty vector had a significant FRET signal at the CIITA pI and MHC II promoters and a weaker signal at the CIITA pIII/IV promoter (Fig. 4D–F), consistent with the pI promoter driving CIITA expression in myeloid cells. Crucially, ectopic expression of NSP5 significantly reduced acetylation at all three promoters, with HDAC2 knockdown restoring promoter acetylation in NSP5-expressing cells (Fig. 4D–F).

### NSP5 targets the CIITA promoter via interactions with IRF3

**Fig. 5.**
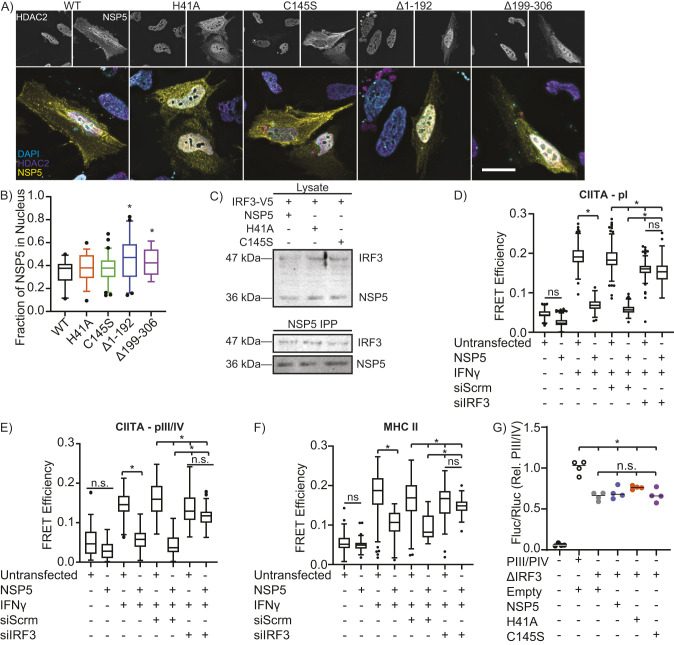
**NSP5 suppresses CIITA promoter activity via IRF3.** (A,B) Fluorescence micrographs (A) and quantification (B) of NSP5 (yellow) and HDAC2 (magenta) nuclear localization in HeLa cells transfected with wild-type (WT) or mutant NSP5. The nucleus has been stained with DAPI (cyan). Scale bar: 10 µm. (C) IRF3 co-immunoprecipitation with wild-type NSP5 and with the H41A and C145S NSP5 catalytic mutants. *n*=3. (D–F) Quantification of FISH-FRET at the CIITA pI (D), CIITA pIII/IV (E) and MHC II (F) promoters in NSP5-transfected A549 cells treated with IFNγ and a non-targeting (siScrm) or IRF3-depleating siRNA (siIRF3). (G) Impact of wild-type (NSP5) versus protease-inactivated H41A and C145S NSP5 mutants on the activity of the wild-type (pIII/IV) or IRF3-binding site deleted (ΔIRF3) CIITA pIII/IV promoter in A549 cells, as quantified by a dual-luciferase assay. Empty, cells are transfected with the empty vector used to express NSP5. Images are representative of a minimum of 30 cells imaged over three independent experiments. Data are representative of (A) or quantify (B–F) three independent experiments, and are plotted as interquartile range with median (box and line) with whiskers representing the 5–95th percentiles (B,D–F) or showing the mean (line, G). *P<0.05; n.s., not significant (Kruskal–Wallis test with Dunn correction compared to WT (B) or the indicated groups (D–G)].

Quantification of the subcellular distribution of HDAC2 and NSP5 point mutants determined that inactivation of the NSP5 catalytic site had no impact on the distribution of either protein (Fig. 5A,B). Although poorly expressed, the rare cells expressing NSP5 deletion mutants lacking the proteolytic (NSP5^Δ1-192^) or B′ (NSP5^Δ199-306^) domains displayed a hyper-nuclear localization for NSP5 compared to wild-type but there was no impact on the distribution of HDAC2, indicating that HDAC2 is unlikely to be the protein used by NSP5 to gain access to the nucleus (Fig. 5A,B). NSP5 has been shown to interact with IRF3, suggesting that within the nucleus NSP5 might deliver HDAC2 to the CIITA promoter via interactions with IRF3 (ENCODE Project Consortium, 2012; Naik et al., 2022; Zuo et al., 2013). Consistent with this hypothesis, IRF3 co-precipitated with NSP5 and this interaction was maintained following inactivation of the NSP5 catalytic site (Fig. 5C; Fig. S4). IFN-γ stimulation increased CIITA pI, pIII/IV and MHC II promoter acetylation in A549 lung epithelial cells, and as expected, NSP5 inhibited acetylation of all three promoters (Fig. 5D–F; Fig. S4). siRNA depletion of IRF3 partially reduced INFγ-induced CIITA promoter acylation, which was not unexpected given that IRF3 is known to bind to the CIITA promoter and contribute to MHC II expression (Zhang et al., 2013, 2020a). Although IRF3 knockdown reduced CIITA promoter acetylation, this knockdown rendered the CIITA promoter refractory to further deacetylation by NSP5 (Fig. 5D–F; Fig. S4). To confirm the specificity of this interaction, a dual luciferase assay was performed using constructs containing the pIII/IV promoter wherein the putative IRF-binding site was deleted (Fig. S1E). As with IRF3 depletion, deletion of this site reduced the INFγ-induced activity of the pIII/IV promoter by ∼30%. However, importantly, this deletion protected the CIITA promoter from NSP5-mediated inactivation, confirming that IRF3 is required for NSP5-mediated suppression of the CIITA promoter (Fig. 5G). These data are consistent with a model wherein NSP5 delivers HDAC2 to the CIITA promoter via interactions with IRF3 bound to the CIITA promoter. Here, HDAC2 deacetylates the CIITA promoter, thus suppressing CIITA expression.

### IRF3 targeting is unique to sarbecoviruses

**Fig. 6.**
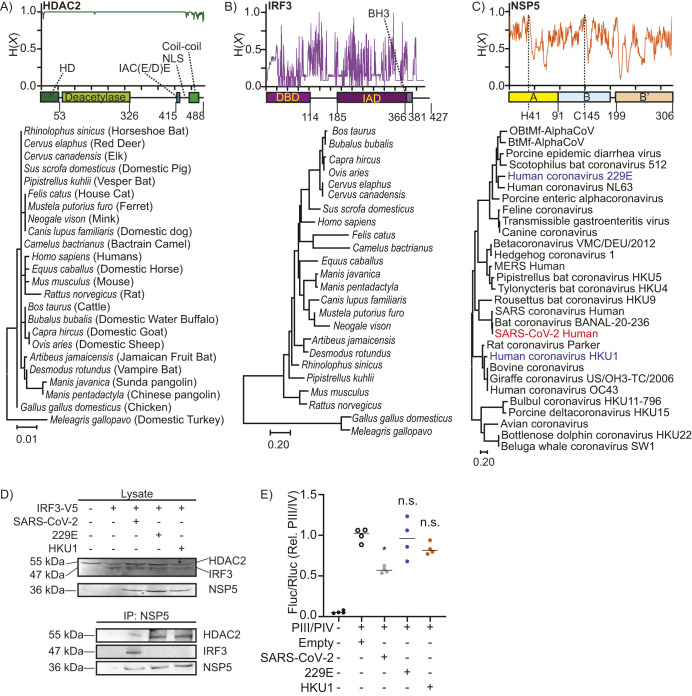
**NSP5 interactions are conserved with HDAC2, but not with IRF3.** (A–C) Domain structure, amino acid conservation [Shannon entropy, H (X)] and phylogenetic trees for HDAC2 (A) and IRF3 (B) across a range of vertebrate species which humans frequently contact or which are possible vectors of SARS-CoV2, and NSP5 (C) across representative coronaviral species. HDAC2 consists of four major domains, a homodimerization (HD) domain, deacetylase domain, an IAC (E/D)E motif, and a coiled-coil domain, which mediate interactions with other transcription factors. IRF3 contains three major domains: a DNA-binding domain (DBD), an IRF-association domain (IAD) and a BH3 domain. NSP5 contains a bipartite protease domain (A/B) with two critical catalytic residues (H41 and C145), and a B′ C-terminal domain, which stabilizes ligands in the protease domain. (D) Co-immunoprecipitation (IP) of endogenous HDAC2 from cells co-transfected with IRF3–V5 and with a FLAG-tagged NSP5 from SARS-CoV-2, from human coronavirus 229E, or from human coronavirus HKU1. Lysate, 5%. Images are representative of three repeats. (E) Dual-luciferase quantification of CIITA promoter pIII/IV in INF-γ-stimulated A549 cells co-transfected with NSP5 from SARS-CoV-2, human coronavirus 229E or human coronavirus HKU1. ‘Empty’ indicates cells co-transfected with the dual-luciferase vectors and the empty version of the vector used to express NSP5. *n*=4, line shows the mean. *P<0.05; n.s., not significant [Kruskal–Wallis test with Dunn correction compared to empty-vector transduced cells].

It was unclear whether the inhibitory effect described above of NSP5 on the CIITA promoter is conserved across the *Coronaviridae* clade. Phylogenetic and amino acid conservation analysis revealed that HDAC2 is strikingly conserved across the vertebrate clade, with most residues completely conserved between humans and a range of vertebrates known to be infected by SARS-CoV-2 or to frequently contact humans (Fig. 6A). In marked contrast, IRF3 was poorly conserved across the same vertebrates (Fig. 6B), and NSP5 was poorly conserved across the major coronavirus clades (Fig. 6C). Although NSP5 is highly divergent across *Coronaviridae*, it is completely conserved within the sarbecovirus subclade of the betacoronaviruses, which includes SARS-CoV, SARS-CoV-2 and bat coronavirus BANAL-20-236 (Fig. 6C). Although the highly conserved nature of HDAC2 would make it a good candidate as a pan-species target of NSP5, the higher diversity of IRF3 might limit the extent to which coronaviruses can utilize IRF3 to target NSP5–HDAC2 complexes to specific promoters. To assess these possibilities, A549 cells were transfected with a V5-tagged IRF3 and with a FLAG-tagged NSP5 from SARS-CoV-2, from the alphacoronavirus 229E or from HKU1 – a member of the embecovirus subclade of the betacoronaviruses (Woo et al., 2010). Interestingly, HDAC2 co-immunoprecipitated with NSP5 from all three coronaviruses, whereas IRF3 only co-immunoprecipitated with NSP5 from SARS-CoV-2 (Fig. 6D). Consistent with this observation, NSP5 from SARS-CoV-2 suppressed promoter activity from the pIII/IV CIITA promoter, but a similar suppression of CIITA was not observed with NSP5 from either 229E or HKU1 (Fig. 6E). Combined, these data indicate that NSP5–HDAC2-mediated epigenetic re-programming is likely conserved across *Coronaviridae*, whereas the IRF3-dependent ‘bridging’ of this NSP5–HDAC2 complex to the CIITA promoter is likely limited to the sarbecoviruses.

## DISCUSSION

**Fig. 7.**
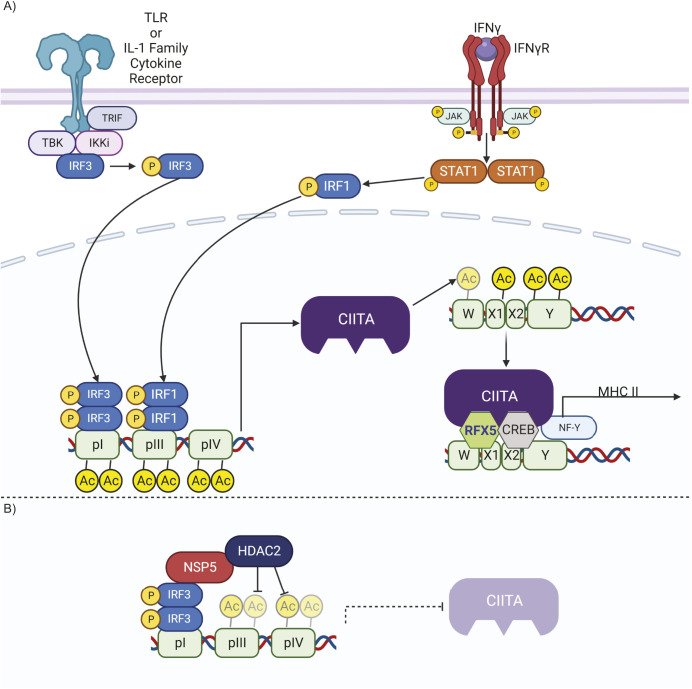
**Model of MHC II transcriptional control and NSP5 activity at the MHC II promoter.** (A) MHC II expression in myeloid cells is induced by a combination of IFN-γ and STAT1 together with TLR or IL-1 family cytokine signaling, which respectively activate IRF1 and IRF3. IRF1 and IRF3 then bind to the CIITA promoter and induce CIITA expression (left). Once synthesized, CIITA directly acetylates histones in the MHC II promoter via its intrinsic acetyltransferase activity. Once the MHC II promoter is acetylated, CIITA, RFX5 and NF-Y form an activating complex on the W, X1, X2 and Y motifs found in the core of the MHC II promoter, inducing expression of MHC II (right). (B) During SARS-CoV-2 infection, NSP5 binds to HDAC2, and via interactions with IRF3, delivers HDAC2 to the CIITA promoter. Here, HDAC2 deacetylates and inactivates the CIITA promoter, thereby suppressing the expression of CIITA, with the resulting loss of CIITA then leading to the cessation of MHC II expression. Figure produced in BioRender.

In this study, we demonstrate that SARS-CoV-2 NSP5 suppresses the expression of CIITA across a range of professional and non-professional APCs, thereby limiting MHC II expression. This suppression of CIITA occurs via a pathway in which NSP5 delivers HDAC2 to the CIITA promoter via interactions between NSP5 and promoter-bound IRF3. HDAC2 then deacetylates histones at the CIITA promoter, decreasing CIITA expression, and thereby blocking MHC II expression (Fig. 7). These findings explain the >50% decrease in MHC II expression observed in the circulating monocytes of individuals with COVID-19, and observed in *in vitro*-infected macrophages (Giamarellos-Bourboulis et al., 2020; Munnur et al., 2021; Wilk et al., 2020). This suppressive mechanism occurred across multiple types of pAPCs (human moDCs, human macrophages and a mouse macrophage cell line) and in human non-professional APCs (type II alveolar epithelial cells). As SARS-CoV-2 infects pAPCs, including DCs, macrophages and B cells, as well as non-professional APCs in the lung, this suppression of MHC II has the potential to dramatically decrease the activation of CD4^+^ T cells in individuals with COVID-19 (Grant et al., 2021; Pontelli et al., 2022; Zhou et al., 2020). Such a loss of CD4^+^ T cell activity likely contributes to the shorter-lived humoral immunity and propensity for reinfection with COVID-19, and might contribute to the aberrant formation of memory and effector CD4^+^ T cell subsets in individuals with severe disease (Ibarrondo et al., 2020; Kaneko et al., 2020; Saichi et al., 2021; Seow et al., 2020; Wiech et al., 2022).

The suppression of MHC I and MHC II expression, or removal of these proteins from the cell surface, is a commonly employed viral immunoevasion strategy. For MHC I, misdirecting or re-internalizing MHC I after its translation are the predominant mechanisms used to limit the immunogenicity of infected cells (van de Weijer et al., 2015). For example, HIV-1 Nef mediates the AP-1-dependent endocytosis of MHC I and its sequestration in a Golgi-proximal compartment, thus limiting cytotoxic T-cell activity against infected cells (Dikeakos et al., 2012; Dirk et al., 2016). Similarly, SARS-CoV-2 utilizes ORF8 to direct MHC I into lysosomes for degradation and uses ORF6 to suppress MHC I expression by downregulating the transcription factor CITA (also known as NLRC5) (Miorin et al., 2020; Zhang et al., 2021). Influenza A and B also downregulate surface MHC I respectively via proteasomal degradation and endocytosis and sequestration in cytosolic vacuoles (Koutsakos et al., 2019). Downregulation of MHC II is rarer, as only viruses that infect APCs have the potential to exhibit this activity. HSV-1 reduces cell surface MHC II by directing MHC II into multivesicular bodies (Temme et al., 2010; Trgovcich et al., 2002). HIV-1 Nef, through the accumulation of αβIi complexes in intracellular vesicles, suppresses the trafficking of peptide-loaded MHC II to the cell surface (Stumptner-Cuvelette et al., 2001). Human cytomegalovirus protein US2 degrades HLA-DR-α and HLA-DM-α chains, whereas the US3 protein competes with the invariant chain for binding to MHC II α/β complexes, thereby restricting MHC II intracellular trafficking (Hegde et al., 2002; Tomazin et al., 1999). Transcriptional downregulation of MHC II is also observed, often through targeting of CIITA. Similar to what we have reported in this study, Kaposi sarcoma-associated herpesvirus targets the IRF3 binding site in the CIITA promoter, but unlike SARS-CoV-2, this virus does so through expressing a viral IRF3 that inhibits CIITA expression (Zuo et al., 2013). Epstein–Barr virus impairs CIITA expression in B cells by suppressing the activity of E47 and PU.1, thereby preventing these transcription factors from binding to the pIII promoter (Lin et al., 2015; van der Stoep et al., 2004).

The extent to which different species of *Coronaviridae* can suppress MHC II is unclear. We found that the NSP5/HDAC2/IRF3-dependent suppression of MHC II was restricted to the betacoronavirus SARS-CoV-2, but given that NSP5 from SARS-CoV and BANAL-20-236 share 100% amino acid identity with SARS-CoV-2 NSP5, this suppression of MHC II expression is expected to be conserved across the sarbecoviruses. This activity was not found in the more distally related betacoronavirus HKU1, nor was it found in alphacoronavirus 229E. Other members of the *Coronaviridae* appear to have independently evolved mechanisms to suppress MHC II expression. The betacoronavirus MERS-CoV also transcriptionally downregulates MHC I and MHC II, but unlike SARS-CoV-2, does so in the presence of elevated CIITA expression (Josset et al., 2013). Alphacoronavirus 229E does not suppress antigen presentation and instead kills infected DCs before they can complete maturation and present antigens (Mesel-Lemoine et al., 2012). As most tested coronaviruses fail to infect pAPCs (Dominguez et al., 2013; Lister, 2009; Woo et al., 2005), and pAPC-infecting coronaviruses are scattered throughout the coronavirus phylogenetic tree, it is likely that the capacity to infect pAPCs and to suppress MHC II expression evolved independently multiple times throughout coronavirus evolution. Such a convergent evolutionary process would account for the lack conservation of MHC II suppression mechanisms across the *Coronaviridae*.

The proteolytic activity of coronaviral NSP5 is known to be important for its immunosuppressive activity. Porcine deltacoronavirus NSP5 suppresses host antiviral IFN signaling by proteolysis of the host antiviral proteins NF-κB essential modulator (Zhu et al., 2017a), STAT2 (Zhu et al., 2017b), and mRNA decapping protein 1a (Zhu et al., 2020b). Previous computational studies also predicted a SARS-CoV-2 NSP5 cleavage site (^380^VQMQ|AIPE^387^) in HDAC2, with cleavage removing an 11.6 kDa C-terminal fragment containing the HDAC2 NLS (Gordon et al., 2020). This cleavage was proposed to reduce HDAC2 localization to the nucleus and thus limit its ability to attenuate inflammatory responses. Surprisingly, we did not observe evidence that NSP5 cleaves HDAC2, with no cleaved HDAC2 detected by immunoblotting or by intramolecular FRET cleavage assays. This is consistent with the work of Naik et al. who observed a similar non-proteolytic interaction of NSP5 with HDAC2 and IRF3, although in contrast, herein we observed a suppressive effect of this interaction on MHC II expression whereas Naik et al. found that this interaction was dispensable for the suppression of IL-6, IL-1β and IFN-β (Naik et al., 2022). Further demonstrating that cleavage is not required for this interaction, the catalytically inactive NSP5^H41A^ and NSP5^C145S^ point mutants maintained their interaction with HDAC2 and IRF3 and had the same suppressive effect on the CIITA and MHC II promoters as wild-type NSP5. Why HDAC2 is not cleaved by NSP5, despite containing an accessible SARS-CoV-2 NSP5 consensus sequence, is unclear. However, non-proteolytic functions of SARS-CoV-2 NSP5 have been reported, including inducing SUMOylation of MAVS to promote inflammation (Li et al., 2021) and suppressing activation of RIG-I by preventing the formation of antiviral stress granules via interactions with G3BP1 (Zheng et al., 2022). Additionally, coronavirus-mediated epigenetic reprogramming has been reported previously, with both SARS-CoV and MERS-CoV engaging in targeted alterations of inhibitory and activating histone modifications across a range of genes (Menachery et al., 2014). Consistent with this observation, we found that SARS-CoV-2 NSP5 selectively inhibited expression of MHC II and CIITA without affecting expression of CD86 and RFX5, or globally suppressing acetylation, with this targeting being driven by the selective delivery of NSP5 to the CIITA promoter via interactions with promoter-binding IRF3.

Although SARS-CoV-2 replicates in the cytosol, at least seven of its proteins have been observed in the nucleus [E, ORF9a, and NSP1, NSP3N, NSP5, NSP6, NSP7, NSP9, NSP10, NSP12, NSP13, NSP14, NSP15 and NSP16 (Zhang et al., 2020b)]. Multiple studies have identified a nuclear pool of SARS-CoV-2 NSP5 (Naik et al., 2022; Zhang et al., 2020b; Zheng et al., 2022), which we determined is formed via an importin-dependent (i.e. ivermectin-sensitive) process. The active transport of NSP5 into the nucleus suggests that NSP5 has specifically evolved to interfere with nuclear processes, and indeed, multiple nuclear activities of SARS-CoV-2 NSP5 have been reported. The epigenetic reprogramming of the CIITA locus we identified is part of a broader pattern of epigenetic modifications that suppress antiviral interferon responses and to promote the cytokine storm (Balnis et al., 2021; Chlamydas et al., 2021). This epigenetic reprogramming occurs, in part, via NSP5 interactions with HDAC2 and BRD2 (Gordon et al., 2020; Luo et al., 2023). In addition to altering nuclear epigenetic marks, SARS-CoV-2 NSP5 directly interferes with transcription via cleavage of POLDIP3, a DNA polymerase δ-interacting protein required for transcription of antiviral interferon-induced genes (Wu et al., 2023). NSP5 also interferes with IRF3-dependent transcription through blocking IRF3 activation downstream of RIG-I signaling, and via modulating its interaction with HDAC2 (Naik et al., 2022; Zheng et al., 2022). A screen for host cell targets of NSP5-mediated proteolysis identified 203 proteins, of which a quarter are nuclear proteins that include DNA polymerases, components of the nuclear pore complex, histones, epigenetic modifiers, and transcription factors (Luo et al., 2023). While it is unknown what effect most of these cleavage events have on host cell function, it is clear that nuclear proteins are an important target for NSP5. While not broadly studied, there is evidence that this nuclear activity is conserved across *Coronaviridae*. Indeed, we observed HDAC2 interactions across three different coronaviruses, while NSP5 from porcine epidemic diarrhea virus, transmissible gastroenteritis virus, and Porcine deltacoronavirus cleave POLDIP3 to suppress interferon-regulated genes (Wu et al., 2023).

HDAC-dependent epigenetic changes drive the cytokine storm and some of the pathology observed in SARS-CoV-2 patients (Chlamydas et al., 2021; Khokhar et al., 2022), and indeed, the extent of these epigenetic changes correlates with disease severity (Balnis et al., 2021; Moura et al., 2021). Consistent with these observations in patients, in model systems HDAC2 is required for the upregulation of many pro-inflammatory molecules in endothelial and myeloid cells undergoing SARS-CoV-2 infection (Bensussen et al., 2021; Shweta and Krishna, 2020; Teodori et al., 2020). These observations suggest that HDAC inhibitors may have clinical benefits for SARS-CoV-2 patients. Indeed, HDAC inhibitors exhibit potent anti-inflammatory effects and therefore may antagonize the inflammatory pathways activated by SARS-CoV-2 and reduce immunopathology in experimentally infected animals (Liu et al., 2020; Ripamonti et al., 2022; Sixto-López and Correa-Basurto, 2022). In addition to regulating inflammation, HDACs also drive the expression of ACE2, with HDAC2 inhibition decreasing ACE2 expression, SARS-CoV-2 entry, and viral replication (Liu et al., 2020; Saiz et al., 2021; Temmam et al., 2022). Moreover, HDAC inhibitors downregulate pro-inflammatory cytokines, reduce lung fibrosis, prevent viral entry into the central nervous system, and decrease neurological damage (P et al., 2021; Ripamonti et al., 2022; Sixto-López and Correa-Basurto, 2022). Thus, HDAC2 inhibition might improve patient outcomes through multiple mechanisms in addition to restoring MHC II expression. Crucially, targeting a host protein would reduce the likelihood of SARS-CoV-2 evolving resistance to this treatment.

The identification of the mechanism used by SARS-CoV-2 to suppress the MHC II antigen presentation pathway provides an important insight into the immunoevasion tactics used by this virus and might help to provide direction for the design of future SARS-CoV-2 vaccines or therapeutics. Interestingly, the suppression of MHC II occurs via the same HDAC-dependent pathway that drives the cytokine storm and enhances viral entry, indicating that targeting this pathway might improve patient outcomes through several mechanisms. Indeed, HDAC2 inhibition has been proposed as a therapeutic approach for COVID-19, with the findings from this study further validating HDAC2 inhibitors as potentially valuable treatments for this disease (P et al., 2021).

## MATERIALS AND METHODS

### Materials

The TGN46–mCherry and KDEL–GFP plasmids were gifts from Dr Sergio Grinstein (Hospital for Sick Children, Toronto, Canada). A549 ACE2 TMPRSS2 cells were a gift from Matthew Miller (McMaster University, Hamilton, Canada). The pMD2.G (plasmid 12259) and pDR8.2 (plasmid 12263) packaging vectors, pcDNA3-myc-CIITA (plasmid 14650), and human IRF3-V5 (plasmid 32713) were purchased from Addgene. All DNA primers and synthesized genes were from IDT (Coralville, Iowa), and the sequences for all primers used in this study can be found in Table S1. Tissue culture medium, fetal bovine serum (FBS) and trypsin were from Wisent (St Bruno, Canada). Recombinant cytokines were from Peprotech (Cranbury, NJ, USA). All cell lines and Lympholyte-poly were from Cedarlane Labs (Burlington, Canada). Polybrene, ivermectin, and 100K Amicon centrifugal filters were purchased from EMD Millipore Corp (USA). The CD14-positive cell selection kit, FcBlock and anti-DYKDDDDK Tag (L5) were from BioLegend (San Diego, California). The #1.5 thickness coverslips and 16% paraformaldehyde (PFA) were from Electron Microscopy Sciences (Hatfield, PA, USA). FDA-traceable PLA filament was purchased from Filaments.ca (Mississauga, Canada). The RNeasy Mini Kit was from Qiagen (Germantown, MD). Permafluor, Versene, WGA–Alexa Fluor 647, DAPI, Hoescht 33258, HALT protease/phosphatase inhibitors, dithiobis[succinimidyl propionate], hygromycin and dithio-bismaleimidoethane, were purchased from Thermo Fisher Scientific (Mississauga, Canada). The suppliers and all antibodies and labeling reagents used in this study can be found in Table S2. The Atto647N NT labeling kit was from Jena Biotech (Jena, Germany). Accell cell-penetrating SMARTpool scrambled and HDAC2-targeting siRNAs were from Horizon Discovery (Cambridge, UK). Instagene, iScript Select cDNA Synthesis Kit, 4–20% SDS-PAGE gels, SsoFast EvaGreen Supermix, and all protein blotting reagents/gels were from Bio-Rad (Mississauga, Canada). *Renilla* luciferase internal control vector pRL-TK and the dual luciferase reporter assay kit were from Promega (Madison, WI, USA). Phusion PCR enzyme, all restriction enzymes, HiFi Gibson Assembly Kit, and T4 DNA ligase were from NEB (Whitby, Canada). The pLVX-zsGreen lentiviral vector and Retro-X Universal Packaging System were purchased from Takara Bio (San Jose, CA, USA). All lab plasticware, PolyJet and GenJet transfection reagents, and DNA isolation kits were from FroggaBio (Concord, Canada), and all laboratory chemicals were from Bioshop (Burlington, Canada).

### Cloning and retroviral packaging

The NSP5 RNA sequence from the Wuhan SARS-CoV-2 strain (Wu et al., 2020), human coronavirus 229E, and human coronavirus HKU1 were synthesized such that a start and stop codon were added to the 5′ and 3′ end of the NSP5 sequence, along with 20 bp of homology to the pLVX-zsGreen vector at the EcoRI restriction site. The resulting NSP5 sequences were cloned into EcoRI-digested pLVX-zsGreen by Gibson assembly. Point mutants were generated by amplifying the entirety of this original vector with phosphorylated primers that incorporate the point mutation in the first base pair of the forward primer, while deletion mutants were generated by amplifying the vector from either side of the desired deletion with phosphorylated primers. After amplification, the parental plasmid was removed by DpnI digestion, and the amplicons were circularized with T4 DNA ligase. NSP5 constructs have been submitted to Addgene. All primers used for RT-qPCR can be found in Table S1. To produce pseudotyped lentivirus containing empty vector or NSP5, 3×10^6^ HEK293T cells (Cedarlane Labs) were grown in 75 cm^2^ tissue culture flasks with DMEM plus 10% FBS, then transfected with PolyJet transfection reagent (500 μl complex containing 4 μg pMD2.G and 10 μg pDR8.2 packaging vectors and 10 μg of pLVX expression vector). Following transfection, cells were incubated at 37°C under 5% CO_2_ for 18 h at which point the medium was exchanged for 8 ml of DMEM supplemented with 10% FBS and returned to the incubator for 48 h. The medium was transferred to a sterile 50 ml conical centrifuge tube and topped up to 20% FBS, then centrifuged at 4000 ***g*** for 5 min, and the supernatant filtered with a 0.2 µm syringe filter into a new 50 ml conical tube. The pseudotyped lentivirus was then concentrated using a 100 kDa centrifugal filter unit at 4000× ***g*** at 4°C for 45 min per 15 ml of filtrate. Concentrated pseudotyped lentivirus was aliquoted and stored at −80°C and thawed at room temperature before use.

### Human macrophage and dendritic cell culture, transduction and siRNA treatment

The collection of blood and cells from healthy donors was approved by the Health Science Research Ethics Board of the University of Western Ontario and was performed following the guidelines of the Tri-Council policy statement on human research. Blood was drawn into heparinized vacuum collection tubes, layered on an equal volume of Lympholyte-poly, and centrifuged at 300 ***g*** for 35 min at 20°C. The top band of peripheral blood mononuclear cells was collected and washed once (300 ***g***, 6 min, 20°C) with phosphate-buffered saline (PBS). For dendritic cell differentiation, a CD14 selection kit was used to isolate monocytes according to the manufacturer's instruction. The selected CD14^+^ cells were cultured in RPMI-1640 plus 10% FBS and 1% antibiotic–antimycotic solution with GM-CSF (100 ng/ml) and IL-4 (100 ng/ml) for 4 days to yield immature monocyte-derived DCs (moDCs). To produce macrophages, selected CD14^+^ cells were cultured in the presence of M-CSF (10 ng/ml) for 6 days. ∼10^6^ moDCs or macrophages were centrifuged with 20 transducing units of lentiviral vectors per cell at 800 ***g*** at 32°C for 90 min with Polybrene (10 μg/ml). After centrifugation, the cells were incubated at 37°C with 5% CO_2_, and 8 h later fresh RPMI-1640 plus 10% FBS and cytokines were added and the cells were incubated for 72 h. For siRNA knockdown, 1 μM of Accell cell-permeant siRNA was added to the cells and incubated at 37°C with 5% CO_2_ for 72-96 h.

### Cell line culture and transfection

HeLa, RAW264.7 and J774.2 cells (Cedarlane Labs) were cultured in DMEM supplemented with 10% FBS, whereas A549 cells were cultured in Ham's F12 supplemented with 10% FBS and were grown at 37°C in a 5% CO_2_ incubator. Cells were split 1:10 upon reaching >80% confluency by either scraping cells into suspension (RAW and J774) or by trypsinization, diluting in fresh medium and replating in a new tissue culture flask. GenJet DNA transfection reagent was used to transfect plasmids into HeLa cells, as per the manufacturer's instructions. Briefly, for each well in a six-well plate, 1 μg of DNA was diluted into 50 μl of serum-free DMEM, followed by 3 μl of GenJet reagent. The resulting mixture was incubated for 10 min at room temperature and then added dropwise to the HeLa cells. Cells were incubated for at least 18 h at 37°C in a 5% CO_2_ before collection. A549 cells were transfected using Lipofectamine 3000, transfecting 1 μg of DNA per well of a 12-well plate, using 2 μl of P2000 and 3 μl Lipofectamine per transfection. A Neon transfection system (Thermo Fisher Scientific) was used to transfect plasmids into RAW264.7 cells according to the manufacturer's instructions. Briefly, 1×10^6^ cells were resuspended in 10 µl of buffer R containing 5 µg of plasmid DNA and electroporated using a single 20 ms pulse at 1680 V. Lentiviral transductions were used for transducing plasmids into J774.2 cells as described above. All cell lines were tested monthly for contamination.

### A549 ACE2 TMPRSS2 cell SARS-CoV-2 infection

A549 ACE2 TMPRSS2 cells were cultured in DMEM supplemented with penicillin (100 U/ml), streptomycin (100 mg/ml), HEPES, L-Glutamine (0.3 mg/ml) and 10% FBS. ACE2 and TMPRSS2 expression was maintained through 700 μg/ml G418 and 800 μg/ml hygromycin supplementation, and cells were cultured at 37°C, 5% CO_2_ and 100% relative humidity. At 1 day before infection, 2×10^4^ A549 ACE2 TMPRSS2 cells were seeded per well in 96-well plates and cultured overnight for a cell monolayer to adhere in G418- and hygromycin-deficient DMEM (37°C, 5% CO_2_). On the day of infection, 10^4^ TCID50/ml SARS-CoV-2 USA-WA1/2020 virus strain was prepared in minimal essential medium (MEM) supplemented with penicillin (100 U/ml), streptomycin (100 mg/ml), HEPES, L-Glutamine (0.3 mg/ml), 0.12% sodium bicarbonate, 2% FBS and 0.24% BSA in a Biosafety Level 3 laboratory (ImPaKT Facility, Western University). The medium was aspirated from the 96-well plates and replaced with a volume corresponding to 500 TCID50 virus per well. Uninfected wells received an equivalent volume of MEM plus 2% FBS lacking virus. All wells were then incubated for 1 h (37°C, 5% CO_2_), at which point virus inoculum or medium was aspirated from all wells and replaced with 100 μl MEM plus 2% FBS, and cultured for a further 72 h.

For immunoblotting, A549 ACE2 TMPRSS2 cell monolayers were washed three times with PBS and collected with minimal versene. Ten wells of a 96-well plate were pooled per independent experiment for both SARS-CoV-2-infected and non-infected conditions. Pooled cell suspensions were centrifuged (500 ***g***, 5 min, room temperature), supernatant was discarded, and cell pellets were lysed for 15 min on ice with 100 µl RIPA buffer supplemented with 1 mM PMSF and Halt protease and phosphatase inhibitor cocktail at the manufacturer's recommended concentration. Cell lysates were clarified (15,000 ***g***, 15 min, room temperature), transferred to a new Eppendorf tube and stored at −80°C. For RNA isolation, monolayers were washed with PBS and 200 μl of TRIzol was added to each well of the 96-well plate. Five wells were pooled per independent experiment for both SARS-CoV-2-infected and non-infected conditions into an Eppendorf tube (1 ml TRIzol) followed by the addition of chloroform (200 µl) and centrifugation (12,000 ***g***, 6 min, room temperature). The aqueous layer was transferred to a new Eppendorf with 1 ml ethanol and placed at −80°C for 20 min. After centrifugation (12,000 ***g***, 25 min, 4°C), the supernatant was discarded and RNA pellet was air dried for 10 min, resuspended in 25 µl RNase free ddH_2_O and stored at −80°C.

### Flow cytometry

HLA-DR and CD86 expression on the surface of moDCs was measured following 72 h transduction with NSP5–ZsGreen or empty-ZsGreen pseudotyped lentivirus and subsequent 24 h stimulation with 100 ng/μl IFN-γ and incubated at 37°C/5% CO_2_. After stimulation, 3×10^5^ cells per condition were washed with PBS and blocked for 30 min on ice with FcBlock. Cells were stained on ice for 30 min using eFluor670-FVD and conjugated primary antibodies as indicated in Table S2. Cells were fixed with 4% PFA in PBS for 15 min then washed with PBS. Expression levels were measured using a FACSCanto (BD), and live moDCs were identified based on FVD-eFluor780 viability dye staining and forward scatter and side scatter profiles. Singlets were gated on the forward area scatter and forward height scatter profiles (Fig. S1A–C). For cell sorting, singlets were gated on the forward area scatter and forward height scatter profiles, then transduced cells were identified by a positive zsGreen signal and this population was sorted into the receiving tube. Flow cytometry data were analyzed using FlowJo (v10.8). All antibodies, dyes and dilutions used for flow cytometry can be found in Table S2.

### Immunoprecipitation and immunoblotting

Before lysis, cells were washed three times with cold PBS. For NSP5–HDAC2 immunoprecipitations, proteins were reversibly cross-linked using the ReCLIP method (Smith et al., 2011). Briefly, cells were incubated for 1 h at room temperature in PBS plus 0.5 mM dithiobis[succinimidyl propionate] and 0.5 mM dithio-bismaleimidoethane. This medium was aspirated, and crosslinking quenched by the addition of 5 mM L-cysteine in 20 mM Tris-HCl pH 7.4 for 10 min at room temperature. All other immunoprecipitations were performed without cross-linking. Cells were suspended in 300 µl of RIPA lysis buffer and 50 µl pre-washed anti-DYKDDDDK Tag (L5) beads, rotating for 1 h. Beads were washed with PBS and immunoprecipitated protein eluted using 0.1 M glycine at pH 2.8, then diluted with 2× Laemmli's buffer with 5% 2-mercaptoethanol, 1 mM PMSF and Halt protease and phosphatase inhibitor cocktail at the manufacturer's recommended concentration. For immunoblotting, cells were lysed with 300 µl RIPA buffer supplemented with 1 mM PMSF and Halt protease and phosphatase inhibitor cocktail at the manufacturer's recommended concentration. Proteins were loaded on 4–15% gradient SDS-PAGE gels and transferred to PVDF membrane. The membrane was blocked for 5 min with EveryBlot Blocking Buffer (Bio-Rad) or 5% BSA in Tris-buffered saline with Tween 20 (TBS-T, 0.9% NaCl, 20 mM Tris-HCl, 0.1% Tween-20, pH 7.4), incubated overnight at 4°C with the desired primary antibodies (Table S2), washed three times for 5 min each time with TBS-T, incubated with appropriate IR700- or IR800-conjugated secondary antibodies (Table S2), 1:2500 dilution, for 1 h at room temperature in TBS-T. The blots were washed three times for 15 min each time in TBS-T and visualized with an Odyssey CLx (LI-COR Biosciences, Lincoln, Nebraska). Densitometry was performed in ImageJ/FIJI (Schindelin et al., 2012; Schneider et al., 2012). Uncropped blots can be found as Fig. S5.

### Half-life determination

The half-life of NSP5 was determined using our established method (Evans et al., 2017). Briefly, HeLa cells were split into six-well plates with 1.5×10^6^ cells/well and transfected with 1 μg/well of wild-type, H41A, C145S, Δ1-192, or Δ199-306 NSP5 using GenJet as per the manufacturer's instructions. After 36 h, the cells were suspended by trypsinization, counted with a hemocytometer, and 2×10^5^ cells/well placed into six wells of a 24-well plate. After 24 h, protein synthesis was halted by the addition of 50 µg/ml cycloheximide, and the cells lysed at 0, 30, 60, 90, 120 and 240 min afterwards. Then, 20 μl of the lysates were immunoblotted with an anti-FLAG antibody as described above, and the quantity of each NSP5 construct was determined at each time point using densitometry. Density was then normalized for each construct to the density of that construct at the 0 min timepoint.

### RT-qPCR

Total RNA was isolated from FACS-sorted cells transduced with either empty or NSP5-expressing lentiviral vectors using an RNeasy Mini Kit as per the manufacturer's instructions. Samples were eluted in 30–50 µl of RNase-free water. RNA concentration and quality were measured using a NanoDrop 1000 Spectrophotometer. cDNA was obtained from total RNA using the iScript Select cDNA Synthesis Kit according to the manufacturer's instructions using an equal amount of starting RNA and an equal mix of the oligo (dT)_20_ primer mixes. RT-qPCR was performed using SsoFast EvaGreen Supermix with an equal amount of starting cDNA. Reactions were run on a QuantStudio 3 Real-Time PCR System for 40 amplification cycles. Relative expression of genes of interest was calculated using the ΔΔCt method, with GAPDH serving as the reference gene.

### Dual-luciferase promoter activity assay

The MHC II HLA-DRA promoter, the CIITA pI promoter and the CIITA pIV promoter (Fig. S1D) were cloned from DNA purified from a human cheek swab using Instagene as per the manufacturer's instructions using the primers in Table S1 and Phusion DNA polymerase and cloned into pGL4.20 digested with EcoRV using Gibson assembly as per the manufacturer's instructions. RAW264.7 cells were seeded at 10^6^ cells/well in a 12-well plate and transfected as indicated with Luc-HLA-DRA, Luc-CIITA pI, Luc-CIITA pIII/IV, *Renilla* luciferase internal control vector pRL-TK, and NSP5–FLAG or pLVX-IRES-ZsGreen using the Neon electroporation system according to the manufacturer's protocol. At 72 h post transfection, cells were lysed with 1× Passive lysis buffer supplemented with EDTA-free HALT protease inhibitor at the manufacturer's recommended concentration. Dual-luciferase assays were performed using a Dual-Luciferase Reporter Assay Kit according to the manufacturer's instructions, with measurements performed on a Cytation 5 luminescence microplate reader. Firefly luciferase readings are presented relative to *Renilla* luciferase readings to account for differences in transfection efficiencies and cell count between samples.

### Immunofluorescence microscopy

Cells of interest were seeded at a density of 1000 cells/mm^2^ into either 18 mm circular coverslips placed into the wells of a 12-well plate, or into the wells of a custom-printed 15-well imaging chamber (Tepperman et al., 2020). Cells were fixed in 4% paraformaldehyde in PBS for 20 min at room temperature. For plasma membrane staining, cells were stained with 5 μg/ml Alexa Fluor 647-conjugated WGA for 10 min at 10°C, then fixed in 4% paraformaldehyde in PEM buffer (80 mM PIPES, 1 mM EGTA and 1 mM MgCl_2_) for 10 min at 37°C. If permeabilization was required, fixed cells were treated with permeabilization buffer (PBS with 0.1% Triton X-100 and 2.5% BSA); otherwise, cells were blocked with antibody buffer (2.5% BSA in PBS). Anti-FLAG, -MHC II or -acetyl-lysine were diluted to the concentration indicated in Table S1 in antibody buffer and incubated with the cells for 1 h. Cells were then washed three times for 15 min each time (3×15 min) with PBS, and then an appropriate secondary antibody (Table S2) was added at a 1:1000 to 1:2500 dilution in antibody buffer for 1 h, followed by washing 3×15 min with PBS. Samples were either imaged immediately or mounted on a slide using Permafluor before imaging. All incubations and washes were performed at room temperature.

All samples were imaged on a Leica DMI6000B equipped with a Hamamatsu ORCA-Flash4 CMOS camera, fast filter wheels equipped with a Chroma Sedat Quad filter set, plus additional FRET filters, operated using Leica LAS-X software. Unless otherwise noted, all cells were imaged using a 100×/1.40 NA objective lens, with *Z*-stacks acquired with 0.4 µm between slices. *Z*-stacks were deconvolved in LAS-X using a 10-iteration Richardson–Lucy deconvolution. Images were exported to ImageJ/FIJI for analysis (Schindelin et al., 2012; Schneider et al., 2012). For colocalization studies, the JaCoP plugin was used to calculate the Manders’ ratio of NSP5–FLAG and nuclear DAPI or Hoechst 33258 staining, or transgene-delineated ER or Golgi markers (Bolte and Cordelières, 2006). To calculate the fraction of NSP5 in the nucleus, a manual region of interest (ROI) was drawn around the nucleus and whole cell, and the integrated intensity of each was measured. The fraction of NSP5 in the nucleus was calculated as the ratio between the integrated intensity of nuclear-localized versus whole-cell NSP5. A minimum of 30 transfected cells were quantified per condition for the colocalization and nuclear ratio assays. FRET and FISH-FRET were quantified as described below. To calculate the mean fluorescence intensity of MHC II in transduced macrophages, the background subtracted channel was thresholded to create a binary mask using the default setting in ImageJ, then a sum slices *Z*-projection was created. A manual ROI was drawn around the whole cell and the integrated intensity was measured. To determine the surface-to-cytosol ratio of MHC II in transduced macrophages, the background subtracted channels for WGA and MHC II were thresholded as described above. Then, the image calculator was used to display all overlapping and non-overlapping pixels, representing MHC II on the cell surface and cytosol, respectively. A summed *Z*-projection was created, and a manual ROI was drawn around the whole cell to measure the integrated density for both surface and cytosolic MHC II. The surface-to-cytosol ratio was calculated as the ratio of the integrated intensity of surface versus cytosolic MHC II signal, and the fraction of total MHC II on the cell surface was calculated as the ratio of the integrated intensity of surface versus whole-cell MHC II signal.

### Intramolecular FRET

DNA consisted of the human HDAC2 gene with flanking BglII and BamHI restriction sites was synthesized and cloned into pmVenus (L68V)-mTurquoise2 (Addgene #60493) such that a mVenus–HDAC2–mTurquoise2 fusion protein was produced. To measure NSP5 proteolysis, a codon-optimized DNA sequence encoding the 20 amino acids of the region of the SARS-CoV-2 ORF1ab transcript that codes for the terminal 10 amino acids of NSP4 and initial 10 amino acids of NSP5 (QTSITSAVLQSGFRKMAFPS) was cloned into the BamHI site of the pmVenus (L68V)-mTurquoise2 vector. This site is a known substrate for NSP5 (Zhao et al., 2022). HeLa cells were then transfected with these constructs with or without NSP5, with mTurquoise2 alone (donor-only sample), with mVenus alone (acceptor-only sample) or with the pmVenus (L68V)-mTurquoise2 vector (positive control). After 24 h of expression, protein translation was inhibited with 100 μM cycloheximide, and the cells were incubated for 6 h to allow NSP5 proteolytic activity to occur in the absence of new protein synthesis. Tiled images of each well were collected, acquiring the donor (mTurquoise2), acceptor (mVenus) and FRET channels at 40× magnification, using the same excitation and camera settings across all samples. FRET efficiency was then calculated using an implementation of the approach of van Rheenen et al. (2004) using a custom-written script in FIJI software (available upon request). In each repeat, the correction values for donor crosstalk (β, donor-only Ida/Idd), donor cross-excitation (α, acceptor-only Idd/Iaa), acceptor cross-excitation (γ, acceptor-only Ida/Iaa), and FRET crosstalk (δ, acceptor-only Idd/Ida), were calculated using donor-only or acceptor-only images and custom-written scripts in FIJI software (available upon request). FRET efficiency (*E*_A_) was then calculated in background subtracted images using the formula:

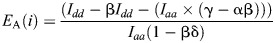
In ImageJ/FIJI, the acceptor-only image was then thresholded, and the ‘Analyze particles’ feature was used to generate separate ROIs for each cell in each image, and these ROIs were used to quantify the FRET signal of each cell. The maximum theoretical FRET efficiency for the mTurquoise2/mVenus FRET pair is 0.3744 (Lambert, 2019).

### FISH-FRET

To measure levels of acetyl-lysine at the MHC II and CIITA promoters, human DNA was purified from a cheek swab using Instagene as per the manufacturer's instructions. 3500 bp amplicons starting before the promoter and ending at the end of the first exon were amplified with Phusion DNA polymerase as per the manufacturer's instructions using the primers from Table S1 (Fig. S1D). Amplicons were gel purified and cloned into EcoRV-digested pBluescript II using a HiFi Assembly Kit as per the manufacturer's instructions. The resulting plasmids were labeled with ATTO647N and fragmented using an ATTO647N NT labeling kit, producing fragments averaging 200 nucleotides. Primary human macrophages or A549 cells were plated into the 7.5 mm wells of a customized imaging chamber (Tepperman et al., 2020), transduced with lentiviral vectors (macrophages) or transfected (A549 cells) with NSP5 or an empty vector, and treated with siRNA as described above. These cells were stained for immuno-FISH as per the protocol of Ye et al. (Ye et al., 2009), including wells that were left unstained, or stained only with the donor (acetyl-lysine-Cy3) or acceptor (FISH probes). Briefly, cells were fixed, permeabilized and immunostained for acetyl-lysine as described above. After labeling a secondary fixation was performed for 10 min with 2% PFA. FISH probes were diluted 1:2500 in hybridization solution (50% formamide, 10% dextran sulfate, 0.3 M NaCl and 30 mM sodium citrate) and denatured at 75°C for 10 min, and then cooled to 37°C. Simultaneously, the cells were incubated at 70°C for 2 min in 70% formamide, 0.3 M NaCl, and 30 mM sodium citrate. The cells were dehydrated by immersing in 75%, 90% and 100% ethanol, at 2 min per immersion, then air dried. The cells were incubated with the denatured FISH probes overnight at 37°C, washed three times for 5 min each time (3×5 min) with 50% formamide, 0.3 M NaCl, and 30 mM sodium citrate at 42°C, then washed 3×5 min with 0.05% Tween 20 in 0.6 M NaCl, and 60 mM sodium citrate. The cells were then washed for 3×5 min in PBS and immediately imaged.

Tiled images of each well were collected, acquiring the zsGreen, donor, acceptor and FRET channels at 40× magnification, using the same excitation and camera settings across all samples. FRET efficiency was then calculated as described above. A trained algorithm in Ilastik (Berg et al., 2019) was used to identify cells based on the acetyl-lysine straining and to classify each cell as zsGreen^+^ (transduced) or zsGreen^−^ (non-transduced); a minimum of 500 FISH-labeled loci were analyzed in each experiment. The resulting classifications were exported to FIJI software where they were used to assign each FISH probe in the image and the corresponding FRET signal to transduced or non-transduced groups. This approach quantifies both histone acetylation and acetylation of promoter-bound transcription factors, with acetylation of transcription factors such as CIITA known to enhance their activity (Spilianakis et al., 2000). The FRET signal in each sample was then normalized to that observed in the scrambled siRNA-treated zsGreen^−^ nuclei. The maximum theoretical FRET efficiency of the Cy3 and ATTO647N FRET pair is 0.3063 (Lambert, 2019).

### NSP5 NLS analysis

The protein sequence of NSP5 was analyzed for the presence of monotonic and bipartite NLSs using the default settings on four different prediction algorithms: cNLS Mapper (https://nls-mapper.iab.keio.ac.jp/cgi-bin/NLS_Mapper_form.cgi), 4 state HMM on NLStradamus (Nguyen Ba et al., 2009), seqNLS (http://mleg.cse.sc.edu/seqNLS/) and in InterProScan (https://www.ebi.ac.uk/interpro/search/sequence/).

### Phylogenetic analysis

Using the protein sequence of human HDAC2 and the NCBI BLASTp tool (https://blast.ncbi.nlm.nih.gov), the protein sequences of HDAC2 from a range of species representing the major vertebrate clades were identified. The same approach, using the protein sequence of NSP5 from SARS-CoV-2 was used to identify NSP5 protein sequences across the four coronavirus genera. These sequences were imported into MEGA XI, and a MUSCLE alignment of the protein sequences was generated (Tamura et al., 2021). Pairwise distances were then calculated using a Poisson model assuming uniform rates across sites, and maximum likelihood trees were generated using a 500-iteration bootstrapping approach. Per-residue conservation was quantified using the Shannon Entropy calculator on the Protein Residue Conservation Prediction server (https://compbio.cs.princeton.edu/conservation/) (Capra and Singh, 2007).

### Statistical analysis

Using GraphPad Prism, a Shapiro–Wilk test was used to determine whether data was parametrically or non-parametrically distributed, and data was then analyzed using an appropriate two-tailed statistical test, as indicated in the figure legends. Parametric data is presented as mean±s.e.m., whereas non-parametric data is presented as box-and-whisker or violin plots with median and quartiles.

## Supplementary Material

10.1242/joces.262172_sup1 Supplementary information
