# Supplementary material for: SARS-CoV-2 NSP5 antagonizes MHC II expression by subverting histone deacetylase 2
Source: J Cell Sci. 2024 May 22;137(10):jcs262172. doi: 10.1242/jcs.262172 (PMC11166459; doi:10.1242/jcs.262172)
Supplement: Supplementary information [file joces-137-262172-s1.pdf]

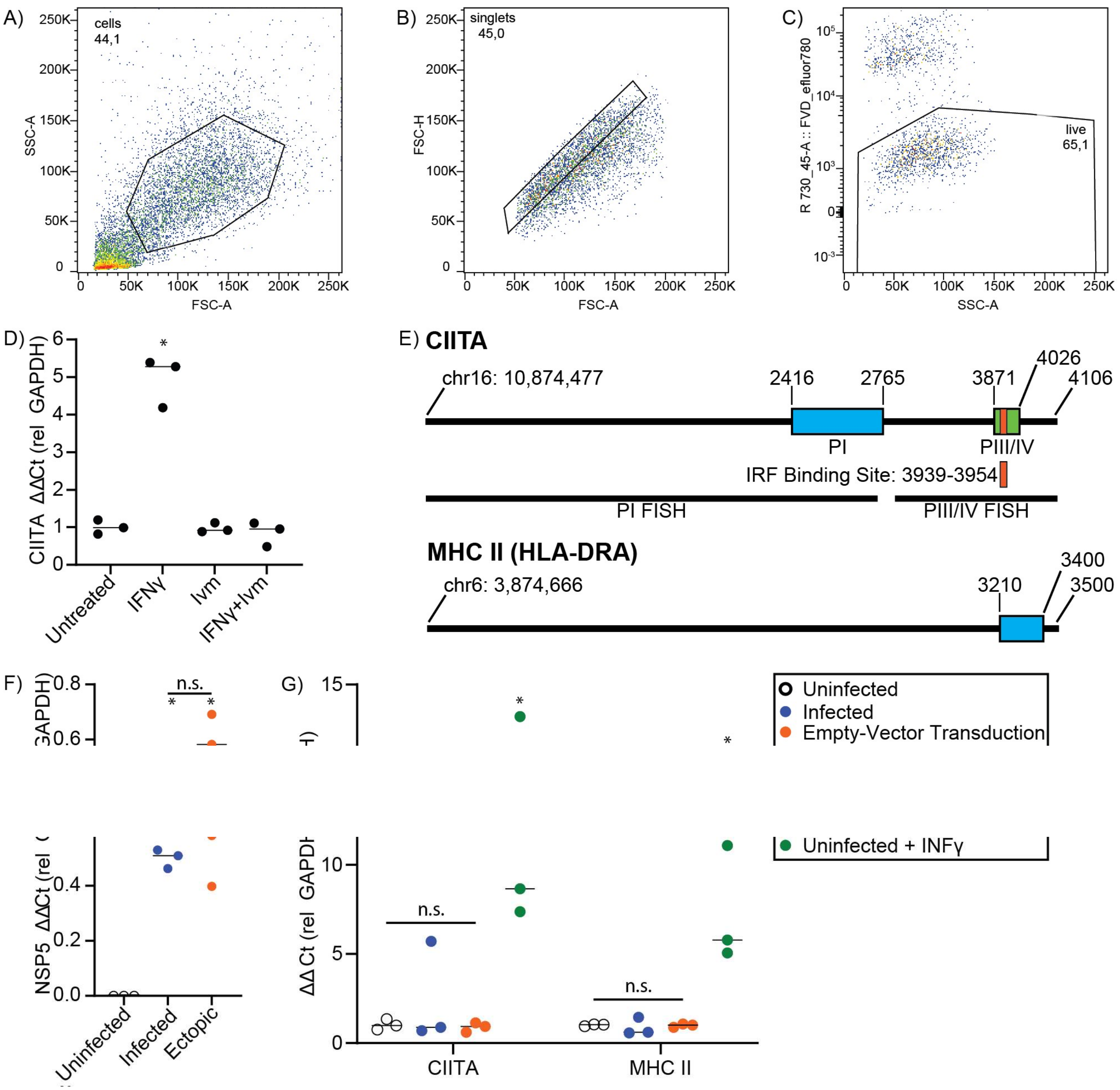
A)
cells
44,1
SSC-A
FSC-A
250K
200K
150K
100K
50K
0
B)
singlets
45,0
FSC-H
FSC-A
C)
live
65,1
R 730_45-A :: FVD_efluor780
SSC-A
D)
CIITA ΔΔCt (rel GAPDH)
Untreated
IFNγ
Ivm
IFNγ+Ivm
E) CIITA
chr16: 10,874,477
2416
2765
3871
4026
4106
PI
PIII/IV
IRF Binding Site: 3939-3954
PI FISH
PIII/IV FISH
MHC II (HLA-DRA)
chr6: 3,874,666
3210
3400
3500
F)
n.s.
NSP5 ΔΔCt (rel GAPDH)
Uninfected
Infected
Ectopic
G)
n.s.
ΔΔCt (rel GAPDH)
CIITA
MHC II
Uninfected
Infected
Empty-Vector Transduction
Uninfected + INFγ

Fig. S1. Detection of MHC II, CIITA, and NSP5 Expression Levels. A-C) Human moDCs were transduced with lentiviral vectors bearing a zsGreen selectable marker and identified by excluding cell debris using the FSC-A/SSC-A scattergrams (A), selecting selected via FSC-A/FSC-H (B), and live moDCs identified by gating on SSC-A and the cell viability dye eFluor670-FVD (C). Up to 10,000 cells were recorded per condition in each experiment. **D)** CIITA expression in macrophages treated with ivermectin (Ivm) and/or INFγ. **E)** Regions cloned for the FISH-FRET and dual-luciferase vectors. For CIITA, two FISH promoters were cloned covering the PI promoter and its 5' region (PI FISH), as well as the region between the end of the PI promoter and 80 bp downstream of the PIII/PIV promoters (PIII/IV FISH). Dual-luciferase vectors containing the PI (blue), PIII/IV (green), and IRF-binding site (orange) deleted PIII/IV promoters were generated. For MHC II, a FISH probe consisting of a 3500 bp region covering from 3200 bp 5' to the HLA-DRA promoter to 100 bp after the promoter was cloned, as was a dual-luciferase vector containing the promoter itself (blue). **F-G)** RT-PCR quantification of NSP5 (F) and MHC II and CIITA expression (G) in A549 cells that were uninfected, infected with the USA-WA1/2020 strain of SARS-CoV-2, transduced with empty or NSP5-expressing lentiviral vectors, or treated with INFγ. Data is expressed as ΔΔCt relative to GAPDH. Data quantifies (D,F,G) or is representative of (A-C) 3 independent experiments. * = $p < 0.05$ compared to uninfected, n.s. = $p > 0.05$ between compared bars, Mann-Whitney U Test.

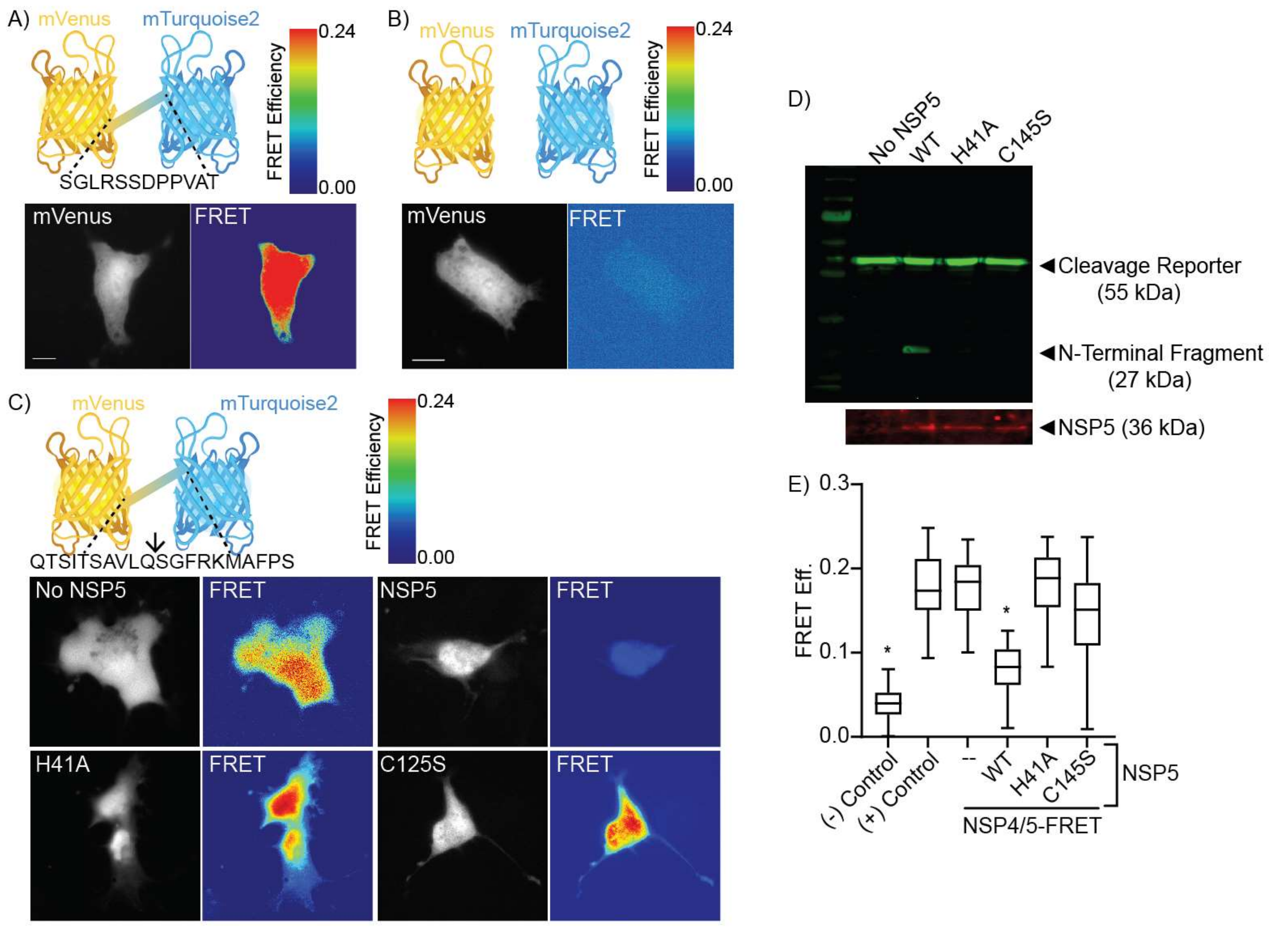

Fig. S2. Quantification of NSP5 Proteolytic Activity. A) Structure (top) and FRET signal (bottom) of the positive FRET control, comprised of mVenus and mTurquoise2 separated by a 12-amino acid linker. **B)** Structure (top) and FRET signal (bottom) of the negative FRET control, comprised of mVenus and mTurquoise2 expressed as separate proteins. **C)** *top:* structure of the NSP5 proteolysis intramolecular FRET probe in which mVenus and mTurquoise2 are linked by the final 10 amino acids of SARS-CoV-2 NSP4 and the first 10 amino acids of SARS-CoV-2 NSP5. The known NSP5 cleavage site is indicated by the arrow. *Bottom:* localization and FRET efficiency of the NSP5 proteolysis probe when expressed in the absence of NSP5 (No NSP5), in the presence of wild-type NSP5 (NSP5), or when expressed with the $NSP5^{H41A}$, or $NSP5^{C145S}$ point mutants. **D)** Quantification of NSP5 proteolysis reporter cleavage by immunoblotting for mVenus showing the expected mass of the uncleaved reporter (55 kDa) and the N-terminal fragment produced by cleavage (27 kDa). **E)** Measurement of NSP5 proteolysis reporter cleavage via quantification of whole-cell FRET. Cells are transfected with the positive or negative control vectors, or with the NSP5 cleavage reporter plus either empty vector (--), wild-type NSP5 (WT), or the $NSP5^{H41A}$ or $NSP5^{C145S}$ point mutants. n = 5, * = $p < 0.05$ compared to the positive control (+) and is presented as interquartile range (box) ± 95% (whiskers), Kruskal-Wallis test with Dunn correction.

Journal of Cell Science • Supplementary information

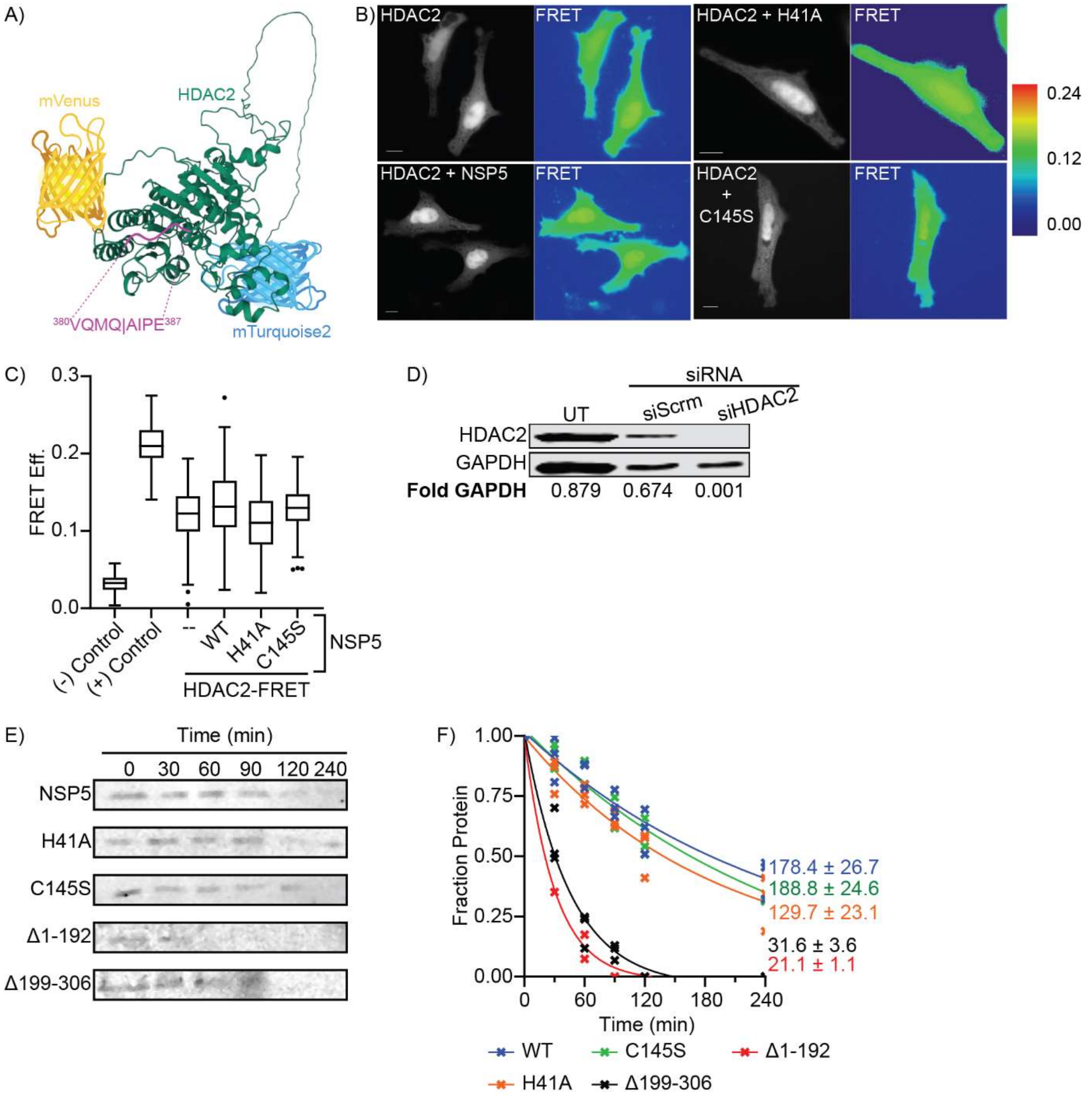

Journal of Cell Science • Supplementary information

Fig. S3. HDAC2 Cleavage and NSP5 Half-Life. A-B) Structure (A) and FRET signal (B) of a HDAC2 intramolecular cleavage FRET probe in which mVenus is fused to the N-terminus, and mTurquoise2 to the C-terminus, of human HDAC2. The putative NSP5 proteolytic site is indicated in purple. **C)** Quantification of HDAC2 cleavage by NSP5 as quantified by whole-cell FRET. Cells are transfected with either the FRET negative control [(-) Control], FRET positive control [(+) Control], or with the HDAC2-FRET reporter plus one of empty NSP5 vector (-), wild-type NSP5 (WT) or with the H41A or C145S NSP5 mutants. **D)** Confirmation of HDAC2 knockdown in cells that are untreated (UT), transfected with a cell-permeant non-targeting siRNA (siScrm), or transfected with a cell-permeant HDAC2-targeting siRNA (siHDAC2). **E)** NSP5 lifespan immunoblots from cells transfected with wild-type NSP5 (NSP5), the $NSP5^{H41A}$ or $NSP5^{C145S}$ point mutants, or the $NSP5^{\Delta 1\text{-}192}$ and $NSP5^{\Delta 199\text{-}306}$ deletion mutants. Protein synthesis was inhibited at t= 0 min with cycloheximide and equal volumes of cell lysate loaded from each indicated timepoint. **F)** Determination of NSP5 half-life by densitometry, with non-linear regression used to calculate the half-life. * = $p < 0.05$ compared to the positive control (+), Kruskal-Wallis test with Dunn correction. Data is presented interquartile range (box) ± 95% (whiskers).

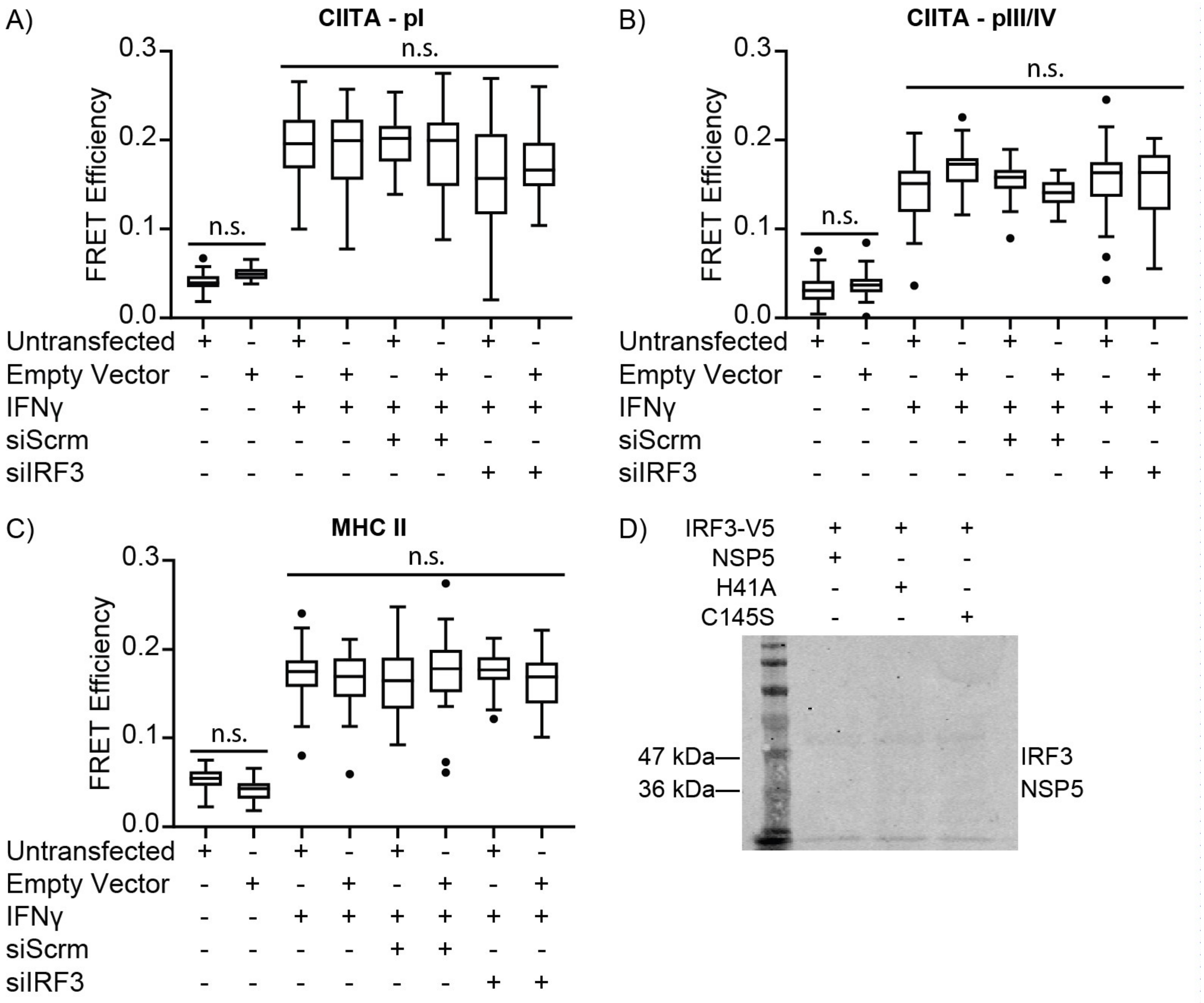

Fig. S4. Control FISH-FRET Data for IRF3 siRNA Knockdown. Quantification of FISH-FRET at the CIITA pI (A), CIITA pII/IV (B) and MHC II (C) promoters in A549 cells co-transfected with an empty vector in lieu of NSP5-expression vectors, and then treated with IFN-γ and a non-targeting (Scrm) or IRF3-depleating siRNA. Data is presented as quartiles, n = 3, * = $p < 0.05$; n.s. = $p > 0.05$ compared to the indicated groups, Kruskal-Wallis test with Dunn correction. Data is presented interquartile range (box) ± 95% (whiskers). **D)** Anti-FLAG + anti-V5 immunoblot of an isotype-control immunoprecipitation from IRF3-V5 and FLAG-NSP5 expressing cells.

Journal of Cell Science • Supplementary information

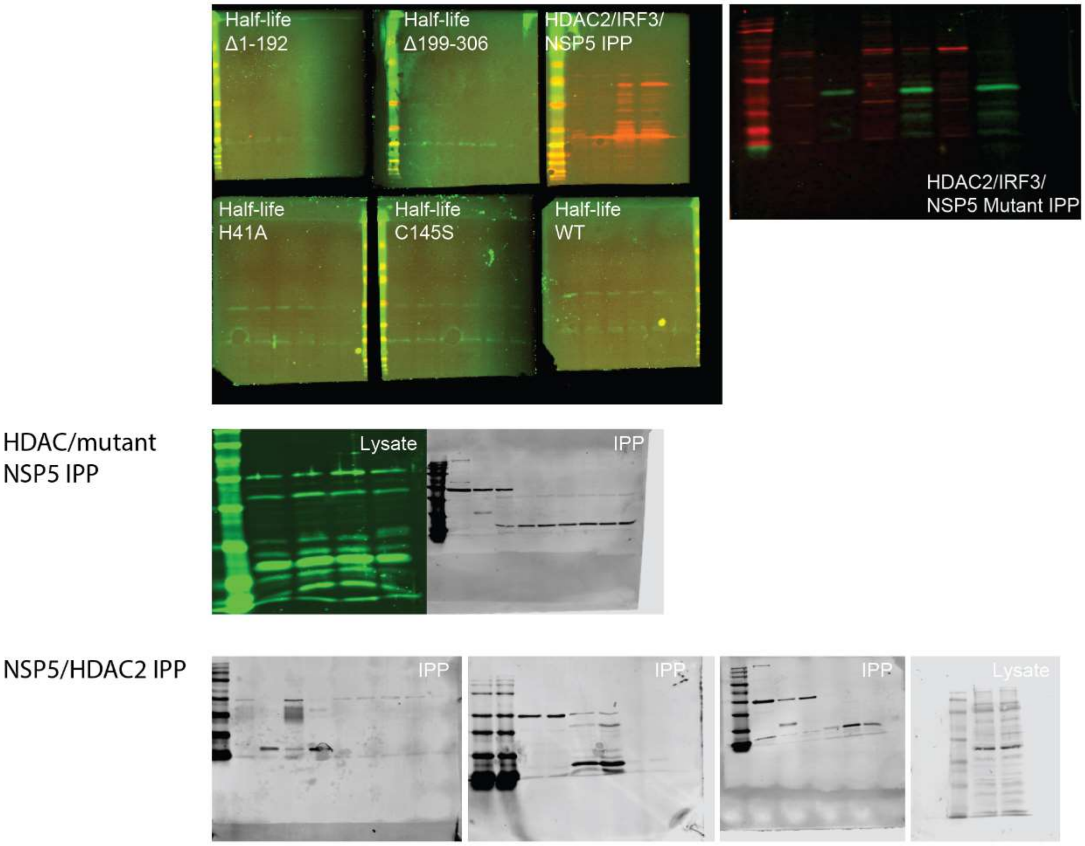

Fig. S5. Western Blot Transparency Figure.

Journal of Cell Science • Supplementary information

Table S1. PCR Primers Used in This Study

Product	Forward Primer	Reverse Primer
MHC II FISH Probe	ACGGT ATCGA TAAGC TTGAT TTTAT TCTGA TAGGG ATCTA TTCCA	CCGGG CTGCA GGAAT TCGAT CTCAG CACCT ACCTT TGATA
CIITA pI FISH Probe	ACGGT ATCGA TAAGC GGCGT GAACC CAGGA GGC	CCGGG CTGCA GGAAT TCGAT ACCTT GGGGC TCTGA CAGGT A
CIITA pIII/IV FISH Probe	ACGGT ATCGA TAAGC TAAAA AGGCC GGGAA AGCAT CTTAA TTTAG CGTG	CCGGG CTGCA GGAAT TCCTG TGGAG CAACC AAGCA CCTAC T
MHC II luciferase reporter	AGCTC GCTAG CCTCG AGGAT TCCGT GATTG ACTAA CAGTC	CGCCG AGGCC AGATC TTGAT GAATA AAAGA AAAGA GAATG TGGG
CIITA luciferase reporter	AGCTC GCTAG CCTCG AGGAT GATAT TGGCA GCTGG CACCA	CGCCG AGGCC AGATC TTGAT CAGCT CAGAA GCACA CAGCC
RFX5 RT-qPCR	TCCTT CAGTT CCATC GTTGA G	TTCAG CTGTC CTCTT GACAC C
CIITA RT-qPCR	CTGAA GGATG TGGAA GACCT GGGAA AG	ACCCT CGTCC CCGAT CTTGT TCTCA CTC
MHC II RT-qPCR	CGAGT TCTAT CTGAA TCCTG	GTTCT GCTGC ATTGC TTTTG C
GAPDH RT-qPCR	TCAAG GCTGA GAACG GGAAG	CGCCC CACTT GATTT TGGAG
NSP5 H41A	P-CTGTG ATCTG CACCT CTGAA GACAT GC	P-CTCTT GGACA GTAAA CTACG TCATC AAGCC A
NSP5 C145S	P-CTGGT AGTGT TGGTT TTAAC ATAGA TTATG ACTGT GT	P-ATGAA CCATT AAGGA ATGAA CCCTT AATAG TGAAA TTG
NSP5$^{\Delta 1\text{-}192}$	P-GCAGC TGGTA CGGAC ACAAC TATTA C	P-CATGA ATTCA CCGGA AATAG ATCCT CTAGT AGAG
NSP5 $^{\Delta 199\text{-}306}$	P-GCATC ACCGG TAGAC TACAA GGACC	P-TGTGT CCGTA CCAGC TGCTT G

Note: "P-" indicates that the 5' end of the primer is phosphorylated.

Journal of Cell Science • Supplementary information

Table S2. Antibodies and Other Staines Used in This Study.

Target	Clone	Species/Isotype	Company	Cat. Number	Concentration*
PE-CD86	BU63	MouseIgG	BioLegend	374202	FC: 0.5 µg/mL
APC-HLA-DR	L243	Mouse IgG	BioLegend	307602	FC: 0.25 µg/mL
FVD-eFluor780	--	--	ThermoFisher	65-0865-14	FC: 1 µL/mL
Human TruStain FcX	--	--	BioLegend	422301	FC: 50 µL/mL
FLAG	6F7	Rat IgG2	Sigma-Aldrich	SAB4200071	IB: 0.5 µg/mL<br>IP: 1.0 µg/mL
V5 Tag	polyclonal	Rabbit	Sigma-Aldrich	SAB1306079	IB: 0.5 µg/mL<br>IP: 1.0 µg/mL
HDAC2	HDAC2-62	Mouse IgG2b	Sigma-Aldrich	H2663	IF: 0.5 µg/mL<br>IB: 0.5 µg/mL<br>IP: 1.0 µg/mL
MHC II, pan-human	RBM1-2967-P1	Rabbit IgG	ThermoFisher	RBM1-2967-P1	FC: 0.5 µg/mL
GAPDH	D16H11	Rabbit IgG	Cell signaling	5174S	IB: 1 µg/mL
α-tubulin	236-10501	Mouse IgG1	ThermoFisher	A11126	IB: 0.5 µg/mL
Acetyl-Lysine	clone 1C6	Mouse IgG	Abcam	ab22550	IF: 1 ng/mL
DAPI	--	--	ThermoFisher	62248	IF: 0.5 µg/mL
Hoechst 33258	--	--	ThermoFisher	H3569	IF: 1 µg/mL
Wheat Germ Agglutinin-Alexa Fluor 647	--	--	ThermoFisher	W32466	IF: 5 µg/mL
Secondary Fluorescent Antibodies and Fab's	--	Various	Jackson Immuno Research Laboratories	Various	*Fab's:*<br>IF: 0.5 - 1 µL/mL<br><br>*Full-Length:*<br>IB: 0.1 µg/mL

** Concentrations used for flow cytometry (FC), immunofluorescence (IF), immunoblotting (IB), or immunoprecipitation (IP).*
